# DNA-directed termination of mammalian RNA polymerase II

**DOI:** 10.1101/gad.351978.124

**Published:** 2024

**Authors:** Lee Davidson, Jérôme O. Rouvière, Rui Sousa-Luís, Takayuki Nojima, Nicholas J. Proudfoot, Torben Heick Jensen, Steven West

**Affiliations:** 1 The Living Systems Institute, University of Exeter, Exeter EX4 4QD, United Kingdom;; 2 Department of Molecular Biology and Genetics, Aarhus University, 8000C Aarhus, Denmark;; 3 Sir William Dunn School of Pathology, Oxford OX1 3RE, United Kingdom;; 4 Medical Institute of Bioregulation, Kyushu University, Higashi-ku, Fukuoka 812-8582, Japan

**Keywords:** exosome, histone, Integrator, RNA polymerase II, transcription termination, Xrn2, snRNA

## Abstract

The best-studied mechanism of eukaryotic RNA polymerase II (RNAPII) transcriptional termination involves polyadenylation site-directed cleavage of the nascent RNA. The RNAPII-associated cleavage product is then degraded by XRN2, dislodging RNAPII from the DNA template. In contrast, prokaryotic RNAP and eukaryotic RNAPIII often terminate directly at T-tracts in the coding DNA strand. Here, we demonstrate a similar and omnipresent capability for mammalian RNAPII. Importantly, this termination mechanism does not require upstream RNA cleavage. Accordingly, T-tract-dependent termination can take place when XRN2 cannot be engaged. We show that T-tracts can terminate snRNA transcription independently of RNA cleavage by the Integrator complex. Importantly, we found genome-wide termination at T-tracts in promoter-proximal regions but not within protein-coding gene bodies. XRN2-dependent termination dominates downstream from protein-coding genes, but the T-tract process is sometimes used. Overall, we demonstrate global DNA-directed attrition of RNAPII transcription, suggesting that RNAPs retain the potential to terminate over T-rich sequences throughout evolution.

## Abstract (precis)

In this study, Davidson et al. report that mammalian RNA polymerase II (RNAPII) can terminate transcription directly at T-tracts, independent of the mechanisms that drive polyadenylation site-directed termination by RNAPII. This DNA-directed reduction in RNAPII-driven transcription, occurring at snRNAs and promoter-proximal regions, highlights an alternative global but conserved mechanism for transcription regulation by RNAPII.

Transcriptional termination of mammalian RNAPII has historically been studied at long protein-coding genes. Here, the process is coupled to 3′ end cleavage and polyadenylation (CPA) of the nascent RNA, which occurs immediately downstream from a polyadenylation site (PAS) (Eaton and West 2020; Rodríguez-Molina et al. 2023). The RNAPII-associated 3′ cleavage product is then degraded 5′ → 3′ by the XRN2 exonuclease, resulting in transcriptional termination (Eaton and West 2020; Eaton et al. 2020; Rodríguez-Molina et al. 2023). This is further facilitated by dephosphorylation of the elongation cofactor SPT5 by the PNUTS-PP1 phosphatase (Cortazar et al. 2019; Eaton et al. 2020). Similar “torpedo” mechanisms are used in lower eukaryotes, prokaryotes, and archaea (Šiková et al. 2020; Wiedermannová and Krásný 2021). Some of these involve endonucleolytic cleavage and exoribonucleolytic activities (Sanders et al. 2020). In other cases, the Rho RNA helicase tracks along the RNAP-associated transcript to elicit termination (Molodtsov et al. 2023).

Other functional mammalian transcripts also undergo 3′ end processing by endonucleolytic cleavage. Small nuclear (sn)RNAs are critical components of the spliceosome, which removes introns from premessenger RNA (pre-mRNA) (Sanford and Caceres 2004). They possess a so-called 3′ box RNA element, which is recognized by the Integrator (INT) complex and cleaved by its resident endonuclease, INTS11, serving as an snRNA maturation factor (Baillat et al. 2005; Guiro and Murphy 2017). INT also associates with phosphatase activity through the interaction between its INTS6 subunit and the PP2A complex (Huang et al. 2020; Zheng et al. 2020). In addition to its role in snRNA processing, INT attenuates transcription in the vicinity of most human RNAPII promoters, and an important function of this activity is to suppress antisense and otherwise cryptic transcription (Elrod et al. 2019; Tatomer et al. 2019; Lykke-Andersen et al. 2021; Stein et al. 2022). INT is suggested to act early in transcription by targeting RNAPII that is paused close to the transcription start site (TSS) (Beckedorff et al. 2020; Stein et al. 2022). Structural and molecular studies support this by showing that INT forms a complex with RNAPII associated with negative elongation factor (NELF), a mediator of promoter-proximal pausing (Stadelmayer et al. 2014; Yamamoto et al. 2014; Fianu et al. 2021). In the structure, the endonuclease active site of INTS11 is open and available, allowing cleavage when the nascent RNA is exposed. Depletion of INT components causes increased transcription beyond snRNA loci, which has been interpreted to reflect a termination mechanism analogous to that at protein-coding genes (Baillat et al. 2005; Stadelmayer et al. 2014; Yamamoto et al. 2014). The model predicts that INTS11-dependent cleavage at the 3′ box exposes an RNAPII-associated RNA to 5′ → 3′ degradation by XRN2. However, XRN2 does not impact either snRNA transcriptional termination or the INT-dependent attenuation of promoter-proximal transcription elsewhere in the genome (Eaton et al. 2018; Elrod et al. 2019). Moreover, although INT depletion causes increased transcription at snRNA loci (Davidson et al. 2020; Lykke-Andersen et al. 2021; Stein et al. 2022), RNA/RNAPII density still returns to background levels further downstream, suggesting the existence of additional termination pathways.

Replication-dependent histone (RDH) genes also produce short (∼500 nt) and intronless RNA. These RNAPII-dependent RNAs are transcribed from RDH gene clusters during S phase (Marzluff and Koreski 2017). Although the CPSF73 endonuclease processes their 3′ ends, RDH RNAs are not polyadenylated, unlike other mRNAs (Dominski et al. 2005; Sun et al. 2020). In further contrast to conventional mRNA 3′ end formation, the RDH processing machinery is recruited by the U7 snRNA rather than via a PAS (Yang et al. 2020). RDH 3′ end formation occurs cotranscriptionally, and there is some evidence that XRN2 can target the RNAPII-associated cleavage product (Sousa-Luís et al. 2021; Cortazar et al. 2022). However, XRN2 has a more limited impact on the termination of RDH transcription compared with that of canonical protein-coding genes (Fong et al. 2015; Eaton et al. 2018). Therefore, additional uncharacterized mechanisms may be used to ensure efficient transcriptional termination.

Beyond the gene classes outlined above, mammalian genomes are pervasively transcribed, yielding a variety of short and largely noncoding (nc) transcripts (Chen et al. 2016). Promoter upstream transcripts (PROMPTs) constitute a prominent example and are transcribed upstream and antisense of most protein-coding TSSs (Core et al. 2008; Preker et al. 2008, 2011; Seila et al. 2008). PROMPT transcription is generally terminated within ∼3 kb of their TSSs, and the resulting RNAs are rapidly degraded in a 3′ → 5′ direction by the nuclear exosome (Preker et al. 2008; Davidson et al. 2019). Similar short and rapidly degraded transcripts are produced in the sense direction of protein-coding genes, which reveals widespread early attrition of RNAPII transcription (Iasillo et al. 2017; Ogami et al. 2017; Davidson et al. 2019). The mechanisms of such termination are beginning to be uncovered and use multiple processes that target immature transcription elongation complexes (Nojima and Proudfoot 2022; Rodríguez-Molina et al. 2023; Wagner et al. 2023). These include INT and the recently described Restrictor complex, which limit the extent of PROMPTs and other cryptic transcription (Austenaa et al. 2021; Estell et al. 2021, 2023; Rouvière et al. 2023). Furthermore, PASs can mediate promoter-proximal transcription termination when the elongation-promoting U1 snRNA is not recruited to pre-mRNA (Kaida et al. 2010; Oh et al. 2017).

A distinguishing feature of most mammalian protein-coding genes versus most other transcription units (TUs) is their length, which frequently exceeds 100 kb due to long intronic sequences. Their full-length transcription therefore requires robust RNAPII elongation complexes that are capable of lengthy transcription of nucleosome-containing templates while simultaneously supporting cotranscriptional RNA processing. The maturation of RNAPII to achieve such elongation competence appears to be a gradual process that occurs over an initial “pausing zone” of ∼3 kb where fully competent RNAPII elongation is not yet established (Fong et al. 2022). Within this zone, RNAPII is prone to prematurely terminate transcription by mechanisms including those mentioned above (Lykke-Andersen et al. 2021). Indeed, exosome depletion stabilizes short (<3 kb) promoter-proximal transcripts at thousands of protein-coding genes (Davidson et al. 2019; Lykke-Andersen et al. 2021). Cyclin-dependent kinase 9 (CDK9) activity is required to release RNAPII held at a promoter-proximal pause (within ∼100 nt of the TSS) but also promotes RNAPII escape from the pausing zone, presumably through its role in phosphorylating the RNAPII C-terminal domain (CTD) and SPT5 (Jonkers and Lis 2015; Core and Adelman 2019; Fujinaga et al. 2023). This accelerates RNAPII transcription through the protein-coding gene body (Jonkers et al. 2014). After crossing the PAS, the transcription complex once again reverts to a slower termination-prone state triggered by the dephosphorylation of SPT5 by PNUTS-PP1 (Parua et al. 2018; Cortazar et al. 2019; Eaton et al. 2020). These observations suggest that RNAPII is most vulnerable to termination when situated promoter-proximally or downstream from a PAS.

DNA sequence plays a direct role in transcriptional termination in many biological contexts. Bacterial RNAP often undergoes transcriptional termination by an intrinsic mechanism (Gusarov and Nudler 1999), which involves the transcription of a hairpin structure followed by a U-tract (representing Ts in the coding DNA strand). This results in a thermodynamically weak dA:rU hybrid within RNAP, favoring termination (Martin and Tinoco 1980). The upstream hairpin aids the process possibly by forward translocation of the RNAP, by shearing of the dA:rU hybrid, or by promoting RNAP conformational change. An analogous mechanism applies for eukaryotic RNAPIII, which terminates at runs of four or more Ts (Arimbasseri et al. 2013, 2014). Recently, the termination of budding yeast RNAPII downstream from the PAS was also shown to involve T-tracts (Han et al. 2023). This was further proposed to aid torpedo-dependent termination by the yeast 5′ → 3′ exonuclease Rat1. AT-rich regions were also suggested to favor mammalian transcriptional termination(White et al. 2013; Vlaming et al. 2022). Importantly, some T-tracts are sufficient to terminate RNAPII in vitro, demonstrating factor-independent activity (Dedrick et al. 1987; Reines et al. 1987; Kerppola and Kane 1990). The generality and location of such transcriptional termination elements remain unexplored in higher eukaryotes.

Here, we report the termination of human RNAPII transcription over T-rich sequences (in the coding DNA strand). This mechanism is widespread promoter-proximally and over short TUs, including snRNA, independent snoRNA, and RDH genes. At snRNA loci, this DNA-directed process is a major mechanism and can be uncoupled from snRNA 3′ end processing by the INT complex. DNA-directed termination is largely independent of the XRN2-dependent torpedo mechanism and predominantly functions promoter-proximally. However, both processes are used downstream from a subset of protein-coding PASs, where the DNA-directed process can terminate transcription that does not efficiently engage the torpedo mechanism. In contrast, T-tracts are largely ineffective within the bodies of long protein-coding genes, suggesting that robust RNAPII elongation complexes acquire resistance to this mechanism. Taken together, we found that immature RNAPII elongation complexes usually located at the beginning and the end of genes can terminate at T-rich elements.

## Results

### What is the role of the INT complex in snRNA transcription?

**Figure 1.**
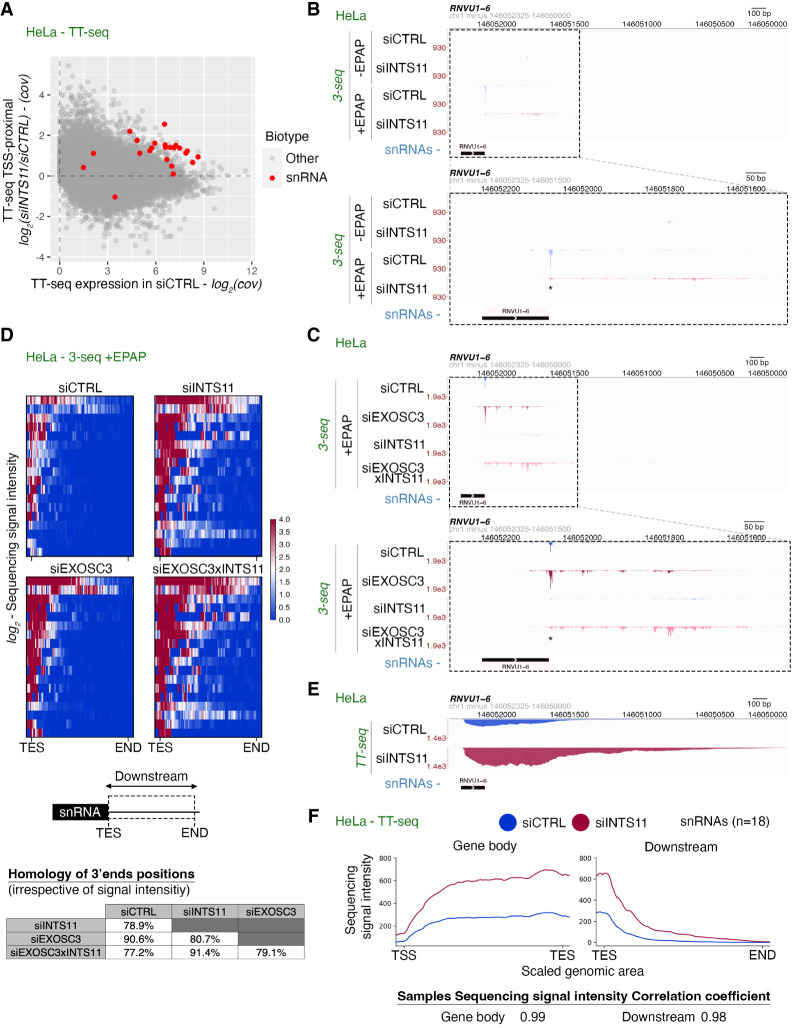
INTS11 is required for snRNA processing but not transcription termination. (*A*) Scatter plot showing the TSS-proximal log_2_ TT-seq signal ratio (siINTS11/siCTRL; *y*-axis) as a function of transcript expression levels (*x*-axis). snRNAs are highlighted in red. The data are from GSE151919 (Lykke-Andersen et al. 2021). (*B*) Genome browser view across and downstream from the *RNVU1-6* TU, displaying RNA 3′ end sequencing (3′-seq) data from siCTRL- and siINTS11-treated HeLa cells (GSE151919) (Lykke-Andersen et al. 2021). Both −EPAP and +EPAP samples are shown. The *top* panel encompasses 2 kb downstream from the *RNVU1-6* body, and the *bottom* panel shows a zoomed-in (dashed box) view of the snRNA-proximal region, with the INTS11 cleavage site indicated by an asterisk. *Y*-axes display reads per kilobase per million mapped reads (RPKM). (*C*) Genome browser view as in *B* but displaying samples from siCTRL-, siINTS11-, siEXOSC3-, or siINTS11 × EXOSC3-treated HeLa cells (GSE151919) (Lykke-Andersen et al. 2021). (*D*) Heat maps of 3′-seq signal coverage downstream from the transcription end sites (TESs) of 18 snRNAs expressed in HeLa cells. Samples were derived from siCTRL, siINTS11, siEXOSC3, or siINTS11 × siEXOSC3 cells (GSE151919) (Lykke-Andersen et al. 2021), and RNA was EPAP-treated. The heat scale represents log_2_ sequencing signal intensity from each depletion condition versus the siCTRL. It highlights similar 3′ end positions between conditions, not the signal intensity differences. The transcription “end” of this region represents the last position of TT-seq read detection following siEXOSC3 depletion (see the Materials and Methods). The accompanying table at the *bottom* displays the percentage homology of the 3′ end positions between each condition. (*E*) Genome browser view as in *B* but displaying TT-seq data from siCTRL- and siINTS11-treated HeLa cells (GSE151919) (Lykke-Andersen et al. 2021). (*F*) Metaplot of TT-seq data from siCTRL- or siINTS11-treated HeLa cells (GSE151919) (Lykke-Andersen et al. 2021) over 18 snRNA loci. The *left* plot shows snRNA gene body signals from the TSS to the TES, and the *right* plot shows signals downstream from the TES. The sequencing signal intensity correlation coefficient demonstrates equivalent increases in read coverage over gene body and downstream regions (see the Materials and Methods).

The mechanism of snRNA transcription termination is assumed to resemble that occurring at protein-coding genes because both use analogous 3′ end processing complexes. Indeed, multiple studies show that INT depletion leads to more transcription downstream from snRNA genes (Baillat et al. 2005; O'Reilly et al. 2014; Davidson et al. 2020; Lykke-Andersen et al. 2021). However, our previous experiments suggested differences because XRN2 did not impact snRNA transcription (Eaton et al. 2018). To better understand what governs the susceptibility of transcription to INT, we reanalyzed our previously generated transient transcriptome sequencing (TT-seq) data from control (siCTRL) and INTS11-depleted (siINTS11-treated) HeLa cells (Lykke-Andersen et al. 2021). For every expressed transcript, regardless of its biotype (*n* = 23,145), we plotted the TSS-proximal log_2_ fold signal change (siINTS11 vs. siCTRL) as a function of the promoter-proximal TT-seq coverage in the siCTRL sample. As previously demonstrated (Lykke-Andersen et al. 2021; Hu et al. 2023), the RNAs with the strongest upregulation following INTS11 depletion tended to derive from lowly expressed TUs (Fig. 1A). However, most snRNAs were clear outliers to this trend by being highly expressed and sensitive to INTS11 loss.

In current models, INT is a productive factor that cleaves and processes pre-snRNAs at their 3′ boxes, which is thought to be coupled to transcriptional termination. This is in contrast to the transcriptional attenuation function of INT found elsewhere, which normally leads to RNA decay rather than maturation (Elrod et al. 2019; Tatomer et al. 2019; Lykke-Andersen et al. 2021) and could be why snRNAs were distinguished from most other INT-sensitive transcripts. Alternatively, INT might also attenuate TSS-proximal snRNA transcription upstream of the 3′ box. The consequent increase in transcription that this would cause might appear as increased readthrough snRNA transcription following INT loss. These considerations necessitated a re-evaluation of INT function(s) in snRNA biogenesis. Hence, we reanalyzed our published sequencing data, representing newly produced RNA 3′ ends (3′-seq) from siCTRL- and siINTS11-treated HeLa cells (Lykke-Andersen et al. 2021). As for the TT-seq data, RNA was metabolically labeled with 4-thiouridine (4sU) and purified by subsequent biotinylation and streptavidin capture (Wu et al. 2020). In contrast to the TT-seq protocol, RNAs were not fragmented before purification. The isolated nascent RNAs represented a mixture of naturally adenylated and nonadenylated transcripts, with the latter including transcripts isolated from the RNAPII active site and possible transcriptional termination products. In our approach, the 3′ ends of cellular RNAs lacking a natural poly(A) tail were detected via their in vitro polyadenylation by *Escherichia coli* poly(A) polymerase (EPAP) (Wu et al. 2020). As 3′ ends of neither mature snRNAs nor their precursors possess a natural poly(A) tail, their detection should be EPAP-dependent. Thus, 3′-seq precisely maps the 3′ ends of RNA deriving from actively transcribing or arrested RNAPII, transcripts released via transcriptional termination, or endonucleolytically cleaved products. The source of 3′ ends can be determined by combining 3′-seq with the depletion of key protein factors.

Illustrating this, the *RNVU1-6* locus displayed almost no signal in samples not subjected to EPAP treatment (−EPAP). However, EPAP treatment (+EPAP) revealed a dominant signal deriving from 3′ box cleavage and lower-level downstream RNA 3′ ends (Fig. 1B, note the bottom panel zoomed-in image of the dashed box). Consistent with INT-dependent snRNA processing, INTS11 depletion abolished the dominant 3′ end. Concomitantly, levels of downstream 3′ ends increased (Fig. 1B, cf. pink and blue signals). These 3′ ends could derive from the active site of RNAPII, signifying an anticipated termination defect triggered by INTS11 depletion. Alternatively, they could correspond to post-transcriptional RNAs released from RNAPII terminating at these sites INTS11-independently. To distinguish these possibilities, we analyzed RNA 3′-seq data from samples where the RNA exosome subunit EXOSC3 had been individually depleted (siEXOSC3) or codepleted with INTS11 (siEXOSC3 × INTS11). Our rationale was that EXOSC3 depletion would preferentially stabilize 3′ ends of post-transcriptional RNAs, whereas nascent 3′ ends would be protected within DNA template-bound RNAPII. Indeed, siEXOSC3 treatment resulted in the accumulation of 3′ ends downstream from the 3′ box (Fig. 1C). Moreover, codepleting INTS11 and EXOSC3 further amplified the signals, suggesting that INTS11 depletion might generally increase snRNA transcription but that the INT complex is not directly required to generate downstream 3′ ends. Although codepleting INTS11 and EXOSC3 increased downstream 3′ end signals, many of their positions were remarkably unchanged between experimental conditions (Fig. 1C, bottom panel). These salient features observed at the *RNVU1-6* locus were evident for other snRNA TUs and more generally observed by meta-analysis (Fig. 1D; Supplemental Fig. S1A). This implies that a significant fraction of snRNA transcriptional termination occurs at specific positions downstream from the snRNA 3′ box via an INT-independent process that releases RNA for degradation by the exosome.

Even though the positioning of RNA 3′ ends downstream from snRNA 3′ boxes was largely unaffected by INTS11 depletion, the complex still affects snRNA transcription because these 3′ ends accumulated upon INTS11 depletion. This would be consistent with previous reports on increased transcription downstream from snRNAs when INT is depleted (Baillat et al. 2005; O'Reilly et al. 2014; Davidson et al. 2020; Lykke-Andersen et al. 2021). To reconcile our findings with these observations, we turned to TT-seq samples performed after siCTRL or siINTS11 treatment of HeLa cells and plotted sequence coverage over and downstream from snRNA loci. In line with the RNA 3′-seq data, the *RNVU1-6* locus displayed an increased TT-seq signal downstream from the 3′ box upon INTS11 depletion (Fig. 1E). Although this could be interpreted as a transcriptional termination defect, there was also an increased TT-seq signal over the *RNVU1-6* gene body, suggesting that INTS11 depletion increases snRNA transcription as suggested above. This was observed for other individual snRNAs (Supplemental Fig. S1B) and by meta-analysis (Fig. 1F; Supplemental Fig. S1C) and was consistent with previously published TT-seq data (Stein et al. 2022). A plausible interpretation of these data is that INTS11 depletion increases snRNA transcription without affecting its natural termination mechanism. In this sense, INT also plays an attenuating role at snRNA TUs analogous to its function throughout the genome and its specific role in 3′ box processing.

### INT-driven snRNA 3′ end processing is cotranscriptional

**Figure 2.**
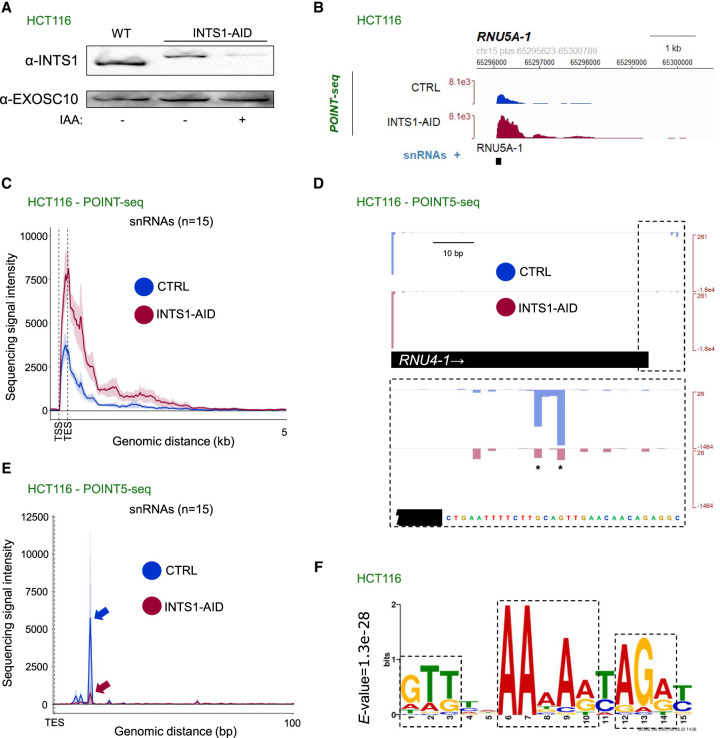
3′ end processing of snRNA precursors occurs cotranscriptionally. (*A*) Western blotting analysis of protein extracts from unmodified (WT) HCT116 cells and *INTS1-AID* HCT116 cells treated (+) or untreated (−) with auxin for 3 h. Note that degron tagging increases the INTS1 molecular weight as expected. EXOSC10 was probed as a loading control. (*B*) Genome browser view across and downstream from the *RNU5A-1* TU, displaying POINT-seq data from *INTS1-AID* HCT116 cells untreated (CTRL) or treated (INTS-AID) with auxin. The *y*-axis sequence signal intensity units are bins per million mapped reads (BPM). (*C*) Metaplot of POINT-seq data of RNA from *INTS1-AID* HCT116 cells untreated (CTRL) or treated (INTS-AID) with auxin. The plot represents 15 snRNAs separated from their neighboring TUs by at least 5 kb. The *x*-axis shows from 0.2 kb upstream of to 5 kb downstream from the annotated snRNA (TSS to TES). The *y*-axis sequence signal intensity units are BPM. (*D*) Genome browser view as in *B* but displaying POINT5-seq data across and downstream from the *RNU4-1* TU. The *top* panel shows the major TSS peak and the minor downstream INT processing site (dashed box). The *bottom* panel displays increased signal resolution around the TES downstream region. The sites of significantly downregulated POINT5 coverage upon INTS1 depletion are marked by asterisks. (*E*) Metaplot as in *C* but displaying POINT5-seq data from snRNA TESs to a region 100 bp downstream. The *y*-axis sequence signal intensity units are BPM. Arrows indicate the major INTS1-sensitive site downstream from the annotated snRNA 3′ end. (*F*) 3′ box consensus element derived from POINT5-seq. INT cleavage occurs immediately upstream of this sequence. The *E*-value represents the probability of encountering the same sequence by chance. See also Supplemental Figure S2B.

Our analyses challenged the long-standing view that snRNA transcriptional termination always requires INT. This model was partly based on the similarity between snRNA 3′ end processing by INT and PAS-dependent mRNA maturation by the CPA complex. Cotranscriptional cleavage near the PAS is a critical feature of the termination mechanism at protein-coding genes (Eaton and West 2020). Regarding 3′ box processing, our data in Figure 1 demonstrated its occurrence on newly transcribed RNA, but the 4sU labeling approach cannot differentiate between cotranscriptional and post-transcriptional INT activity. To ask whether INT processes 3′ box-containing RNA cotranscriptionally, we therefore used POINT5-seq (Sousa-Luís et al. 2021). Here, chromatin-associated RNAPII is immunopurified with its nascent transcript. The 5′ ends of these RNAs are then mapped with single-nucleotide resolution via strand-switching reverse transcription followed by RNA-seq. In parallel, we performed POINT-seq analysis, where RNAs from chromatin-associated RNAPII were sequenced in their entirety to provide an overall picture of nascent transcription. These analyses were performed in HCT116 cells in which the INT backbone subunit INTS1 was tagged with an auxin-inducible degron (AID), allowing its depletion within 3 h of auxin (IAA) addition (Fig. 2A). This rapid depletion provided confidence that any observed effect was a primary consequence of INTS1 loss.

INTS1-AID depletion caused an elevated POINT-seq signal over the snRNA gene body and throughout the downstream region, exemplified by the *RNU5A-1* TU (Fig. 2B). However, the POINT-seq signal eventually returned to background levels even when INTS1 was depleted. Meta-analysis confirmed the generality of this observation for other snRNAs (Fig. 2C) while also demonstrating that although the INTS1-AID depletion curve displayed a higher signal than the CTRL curve, they both had a similar shape. This agreed with our conclusion from Figure 1 that INT loss increases snRNA transcription but may not affect the utilization of major transcriptional termination sites downstream from the TU. The data also demonstrated consistency between siRNA-mediated depletion of INTS11 from HeLa cells and AID-mediated depletion of INTS1 from HCT116 cells.

Next, we used the POINT5-seq data to assess whether snRNA 3′ end processing is cotranscriptional. At the exemplary *RNU4-1* locus, POINT5-seq revealed two nascent 5′ end signals: a major one at the TSS and another minor doublet just beyond the annotated gene body (Fig. 2D, note dashed box highlighting the minor doublet). We assumed the latter represents 3′ box processing by INT because it was reduced upon INTS1 depletion. An additional example of cotranscriptional 3′ box processing by INT was provided by the *RNU12* locus (Supplemental Fig. S2A) and was generally evident at other snRNA loci (Fig. 2E). These INT cleavage sites were situated downstream from the annotated snRNA 3′ end, consistent with previous findings that mature snRNA 3′ ends are formed by TOE1-mediated 3′ → 5′ trimming of a remaining short 3′ extension (Lardelli and Lykke-Andersen 2020). Thus, like PAS cleavage, 3′ box processing is cotranscriptional. However, unlike PAS cleavage, eliminating 3′ box processing does not prevent transcriptional termination.

By performing a MEME analysis of the 30 nt immediately downstream from snRNA TESs, we identified a 3′ box consensus element downstream from the detected INT cleavage sites (Fig. 2F; Supplemental Fig. S2B). To test its effectiveness as an snRNA 3′ end processing signal, we used a previously established GFP reporter assay (Albrecht and Wagner 2012; Albrecht et al. 2018). In this system, 3′ end box processing precludes GFP expression, which only occurs when processing fails (Supplemental Fig. S2C). By this approach, the consensus 3′ box proved an efficient snRNA processing element (Supplemental Fig. S2D). Interestingly, mutation of its most highly conserved nucleotides had only modest effects on snRNA processing, implying that the 3′ box is more resistant to mutation than the PAS, which can be inactivated by single nucleotide substitutions (Proudfoot 2011). We then asked whether similar 3′ box sequences might be active in other promoter-proximal regions. If this was the case, any POINT5-seq signal should be reduced by INTS1 depletion. However, despite being highly active at snRNA loci, the 3′ box consensus remained inactive in other promoter-proximal contexts (Supplemental Fig. S2E). We presume that 3′ box processing requires an snRNA-specific factor or feature, as suggested by prior work showing that snRNA maturation requires transcription from an snRNA promoter (Hernandez and Weiner 1986). Overall, we conclude that 3′ box processing is cotranscriptional and specific for snRNAs.

### The 3′ box elicits snRNA processing but is dispensable for transcription termination

**Figure 3.**
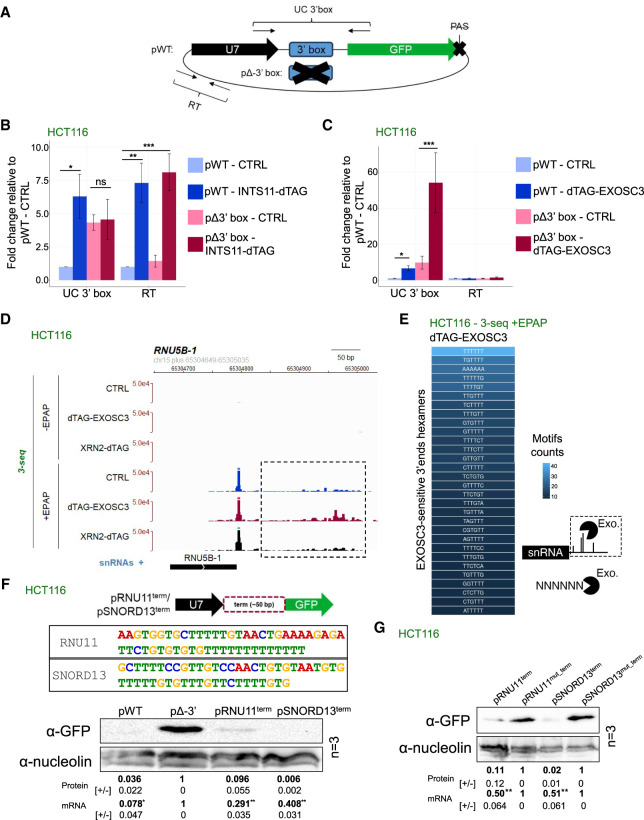
Termination of snRNA transcription is 3′ box-independent. (*A*) Schematic representation of the pWT and pΔ3′ box reporter plasmids. The U7 snRNA sequence is followed by its 3′ box (or its deletion) and a GFP gene with a mutated PAS. Arrow pairs denote the two amplicons (“UC 3′ box” and “RT”) used for qRT-PCR analysis. (*B*) qRT-PCR analysis of pWT or pΔ3′ box reporter expression from *INTS11-dTAG* HCT116 cells untreated (CTRL) or treated (INTS11-dTAG) for 4 h with dTAGv-1. RNA spanning the 3′ box or from the downstream region was measured by “UC 3′ box” and “RT” amplicons, respectively. Measured RNA quantities were normalized to levels of GAPDH RNA. Mean fold change values were calculated by comparative quantitation versus pWT CTRL. *n* = 4. Error bars indicate standard error of the mean (SEM). (*) *P* ≤ 0.05, (**) *P* ≤ 0.01, (***) *P* ≤ 0.001. (*C*) As in *B* but performed in *dTAG-EXOSC3* HCT116 cells. *n* = 4. Error bars indicate SEM. (*) *P* ≤ 0.05, (***) *P* ≤ 0.001. (*D*) Genome browser view of RNA 3′-seq signal over the *RNU5B-1* locus and deriving from ±EPAP-treated RNA from HCT116 (CTRL), *dTAG-EXOSC3*, or *XRN2-dTAG* cells treated with dTAGv-1 for 4 h. The dashed box highlights a region with EXOSC3-sensitive 3′ ends. (*E*) Sequence composition of the most common hexameric motifs at the 3′ terminus of 3′-seq reads (+EPAP) found ≤4 kb downstream from snRNA TESs and stabilized by log_2_ change ≥1 following EXOSC3 depletion from dTAG-EXOSC3 cells versus HCT116 CTRL cells (see schematic). Motifs are shown as the coding DNA strand equivalent. The heat scale shows the motif count. (*F*, *top* panel) Schematic representation of used reporter constructs and the sequences of the inserted *RNU11* and *SNORD13* downstream elements. (*Bottom* panel) Western blotting analysis of GFP expression following transfection of HCT116 cells with the pWT-, pΔ3′ box-, pRNU11^term^-, or pSNORD13^term^-containing reporter constructs. Quantifications *below* the Western membrane show relative GFP expression levels relative to those of the pΔ3′ box reporter. *n* = 3 (±values = SEM) following normalization to nucleolin protein levels. GFP mRNA levels are displayed as determined by qRT-PCR. *n* = 3. (*) *P* < 0.05, (**) *P* < 0.01. (*G*) Western blotting and qRT-PCR analysis as in *F* but of the pRNU11^term^ and pSNORD13^term^ constructs and their mutated derivatives, in which all Ts were substituted for As.

Our above analyses suggested that INT is not critical for snRNA transcription termination. Still, 3′ box cleavage is cotranscriptional and strongly impacts GFP levels in our reporter assay. We therefore more directly addressed whether the 3′ box plays any role in snRNA transcription. Accordingly, we generated two reporter plasmids based on the design from Supplemental Figure S2C—one containing the U7 snRNA and its 3′ box (pWT) and another from which the 3′ box had been deleted (pΔ3′box) (Fig. 3A). Both plasmids contained the downstream GFP gene, the PAS of which we removed to isolate any effect of 3′ box mutation on transcription termination. pWT and pΔ3′ box plasmids were transfected into our recently described *INTS11-dTAG* cell line (Eaton et al. 2024), and following depletion of INTS11, qRT-PCR analysis was performed to assay RNA spanning the 3′ box (UC 3′ box amplicon) or at a downstream readthrough position (RT amplicon) (Fig. 3A, see positions of used amplicons). Deletion of the 3′ box resulted in significantly more RNA spanning the 3′ box region, consistent with its processing activity (Fig. 3B, left panel, cf. “UC 3′ box” light-blue and pink columns). INTS11 depletion also increased UC 3′ box RNA levels but only for pWT, demonstrating that 3′ box deletion eliminated both 3′ end processing activity and INTS11 sensitivity. Even so, readthrough RNA levels were unchanged between pWT and pΔ3′ box constructs in CTRL samples (Fig. 3B, cf. “RT” light-blue and pink columns), suggesting that the 3′ box is unnecessary for transcriptional termination. This is in contrast to the widely observed abolition of transcriptional termination on similar protein-coding reporter plasmids containing mutated PASs (Whitelaw and Proudfoot 1986; Dye and Proudfoot 1999, 2001). Interestingly, INTS11 depletion significantly increased RT RNA levels from both constructs, demonstrating that the INT complex impacts reporter transcription in a 3′ box-independent manner. This reconciles our described INT-independent snRNA transcription termination and the increased snRNA transcription downstream from the TES widely seen following INT loss (including the TT-seq and POINT-seq data above). We note that although INTS11 depletion did not enhance RNA levels measured by the UC 3′ box amplicon in pΔ3′ box samples, these levels were already high due to the lack of 3′ box cleavage. We therefore suggest that snRNA transcriptional readthrough following INTS11 depletion derives from increased transcription due to a diminished attenuating activity upstream of the 3′ box.

Our results from Figure 1 showed that the exosome degrades the products of an INT-independent snRNA transcriptional termination process. This collectively predicted that snRNA termination will continue to generate exosome substrates even when a 3′ box is absent. To test this, we transfected the pWT and pΔ3′ box plasmids into *dTAG-EXOSC3* HCT116 cells, allowing rapid exosome inactivation (Supplemental Fig. S3A). Unlike the INTS11 depletion, which only affected UC 3′ box amplicon levels when the 3′ box was present, exosome depletion substantially enhanced the amount of RNA spanning the 3′ box region for both pWT and pΔ3′ box constructs (Fig. 3C, “UC 3′ box” columns). In contrast, exosome depletion did not affect RT RNA expression from either construct, presumably because the 3′ box-independent termination process remained fully operational and because the exosome has no direct effect on the transcription attenuation process. Therefore, a 3′ box-independent termination mechanism generates snRNA precursors, which are substrates for the exosome. We suggest that cotranscriptional 3′ box processing by the INT complex insulates the mature snRNA from this degradation initiating from downstream termination sites. Supporting this notion, published photoactivatable ribonucleoside-enhanced cross-linking and immunoprecipitation (PAR-CLIP) data of the exosome ribonuclease DIS3 (Szczepińska et al. 2015) demonstrated its elevated binding to regions downstream from the snRNA 3′ box (Supplemental Fig. S3B).

We previously detected signs of snRNA transcription termination at T-rich elements (Davidson et al. 2020). To explore this systematically, we generated further RNA 3′-seq data to map the 3′ ends of newly produced transcripts but using dTAG degron HCT116 cells for rapid EXOSC3 depletion or, to test any contribution of 5′ → 3′ degradation, XRN2 (Supplemental Fig. S3A). As expected, the *RNU5B-1* locus showed no read coverage in the −EPAP conditions (Fig. 3D, top panels). In contrast, +EPAP samples displayed the products of 3′ box cleavage (Fig. 3D, +EPAP panels). Moreover, numerous downstream 3′ ends were revealed and were stabilized by EXOSC3 loss. This finding is consistent with results from Figure 1C showing reproducibility between data obtained following siRNA-mediated depletion of EXOSC3 from HeLa cells and degron depletion of EXOSC3 from HCT116 cells. XRN2 depletion did not impact positions or levels of downstream 3′ ends, in line with its inability to terminate snRNA transcription (Eaton et al. 2018).

To assess the contribution of DNA/RNA sequences to snRNA transcription termination in an unbiased manner, we extracted the most common 3′ terminal hexamers that were upregulated by log_2_ change ≥1 in +EPAP 3′-seq samples from dTAG-treated dTAG-EXOSC3 versus CTRL HCT116 cells. We surmised that these exosome-degraded transcripts were terminated RNAs, as indicated by our experiments in Figure 1, and that nucleotides at their 3′ ends should represent any functional DNA sequence motifs. To be conservative, we searched for hexameric sequences knowing that four or more Ts are sufficient to induce DNA-directed transcriptional termination of RNAPIII (Arimbasseri et al. 2013). Plotting sequences as their DNA coding strand equivalent revealed T6 as the most enriched hexamer as well as several additional T-rich elements (Fig. 3E). Importantly, this did not simply reflect a T bias of the underlying DNA strand because the percentage of exosome-sensitive 3′ ends terminating at coding strand Ts exceeded the percentage of Ts in the same genomic sequence (Supplemental Fig. S3C).

To test whether naturally occurring T-rich elements could act as terminators when transplanted into reporter DNA, we replaced the 3′ box within our U7-GFP reporter (retaining the GFP PAS in this case) with ∼50 nt of T-rich elements from downstream from *RNU11* or *SNORD13* sn/snoRNA TESs. These contained visible clusters of EXOSC3-sensitive 3′ ends (Supplemental Fig. S3D). The resulting plasmids were transfected into HCT116 cells along with the pWT and pΔ3′ box reporter controls, and GFP protein and mRNA levels were assayed by Western blotting and qRT-PCR, respectively. As expected, protein and mRNA levels were low for pWT and high for pΔ3′ box constructs (Fig. 3F). Replacing the 3′ box with *SNORD13* or *RNU11* sequence strongly reduced GFP expression, suggesting that these sequences terminate transcription. We then substituted all Ts within the cloned elements with As, which resulted in the recovery of GFP expression (Fig. 3G). Interestingly, termination activity was retained after substituting T-tracts for AT-tracts within the SNORD13 terminator (Supplemental Fig. S3E). Last, a homopolymeric T-tract displayed some termination capacity in this reporter (Supplemental Fig. S3F). Thus, natural and artificial T-rich elements promote transcriptional termination.

An alternative explanation for our hypothesis—that exosome-sensitive 3′ ends are generated by transcriptional termination—could be that they are formed by endonucleolytic cleavage. However, arguing against this possibility, our POINT5-seq analysis did not detect INT-dependent cleavage activity at T-rich elements downstream from snRNAs (Supplemental Fig. S4A). Consequently, we propose that termination occurs directly over T-rich sequences constituting a DNA-directed mechanism. Although INT depletion increases transcription downstream from snRNAs, this can be accounted for by its attenuation of snRNA transcription. Interestingly, this attenuation function of INT is not associated with detectable POINT5 peaks over snRNA gene bodies or in other promoter-proximal regions, which suggests that 3′ end box processing is uniquely well defined (Supplemental Fig. S4B,C). Nevertheless, some RNAPII complexes read through T-rich terminators when INTS11 is depleted, illustrating an indirect effect due to relieved transcriptional attenuation (Supplemental Fig. S4D,E). Taking all of our data together, we suggest a revised model for Integrator function at snRNA loci, where the complex exercises a dual role in attenuating snRNA transcription as well as processing the nascent RNA at the 3′ box (Supplemental Fig. S4F).

### Genome-wide transcriptional termination at T-rich elements

**Figure 4.**
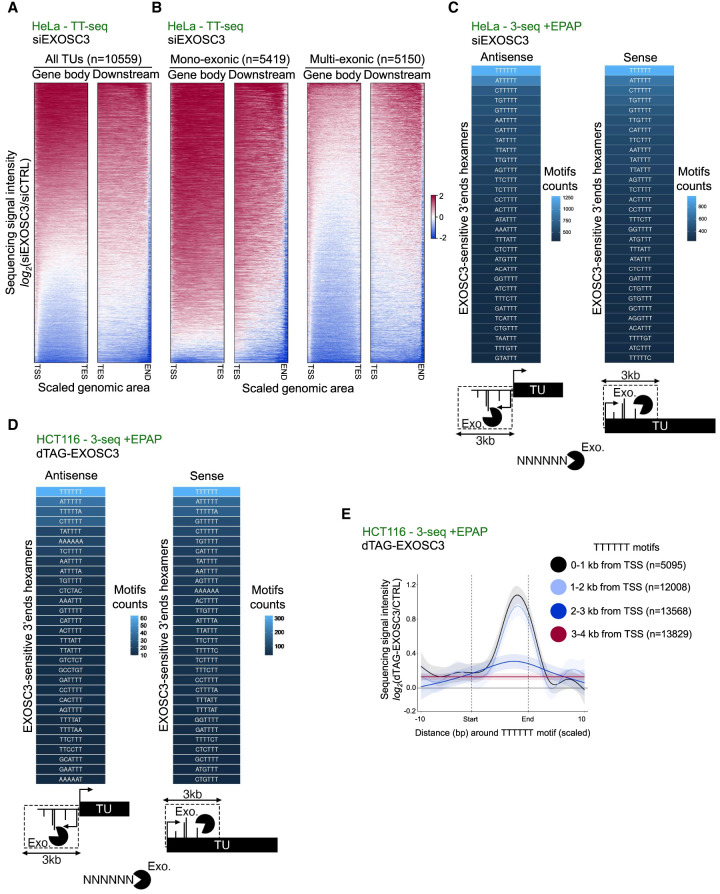
Global transcriptional termination at T-rich elements. (*A*) Heat maps showing TT-seq data from siCTRL- and siEXOSC3-treated HeLa cell RNA samples (GSE151919) (Lykke-Andersen et al. 2021). The *left* heat map shows the log_2_ change in sequencing signal intensity (siEXOSC3 vs. siCTRL) over the gene body region (TSS to TES) of all TUs (*n* = 10,559), and the *right* heat map shows the same analysis but for the area downstream from the TES. (*B*) Heat maps as in *A* but showing the gene body and TES downstream regions of monoexonic (*left* two maps) and multiexonic (*right* two maps) TUs. (*C*) Sequence composition of the most common hexameric motifs at the 3′ end of 3′-seq (+EPAP) reads over regions within 3 kb sense or antisense of TSSs and stabilized by log_2_ change ≥1 following siEXOSC3 versus siCTRL treatment of HeLa cells. Note that motifs are shown as the coding DNA strand sequence. The heat scale shows motif counts. Analyses were derived from GSE151919 (Lykke-Andersen et al. 2021). (*D*) Analysis as in *C* but in dTAG-treated *dTAG-EXOSC3* HCT116 cells versus CTRL HCT116 cells. (*E*) Meta-analysis of +EPAP 3′-seq coverage over motifs of T6 or greater within 0–1, 1–2, 2–3, or 3–4 kb from multiexonic TU TSSs. The *x*-axis shows this region (start–end), including 10 nt upstream and downstream. The *y*-axis displays the log_2_ change in signal sequence intensity in dTAG-treated *dTAG-EXOSC3* or *XRN2-dTAG* HCT116 cells versus HCT116 CTRL cells.

Having established snRNA transcription termination at T-rich elements, we wondered whether such termination might be more widespread. Beyond pre-snRNAs, many transcripts are exosome-sensitive, which might in part be explained by termination over T-stretches. Hence, we broadly assessed the exosome sensitivity of newly synthesized RNA, reasoning that this would predict such DNA-directed termination. We used TT-seq from siCTRL- or siEXOSC3-treated HeLa cells (Lykke-Andersen et al. 2021) and plotted the log_2_ fold change in signal for siEXOSC3 versus siCTRL samples over the bodies of all TUs with TT-seq signal detected downstream from their annotated TESs. This analysis revealed the expected production of exosome-sensitive transcripts (Fig. 4A). We previously demonstrated that RNAs deriving from monoexonic TUs are more exosome-sensitive than RNAs from multiexonic TUs (Lykke-Andersen et al. 2021). Indeed, stratifying the TT-seq analysis by mono- and multiexonic TUs recapitulated this finding (Fig. 4B). The production of unstable RNA from gene bodies and downstream regions of monoexonic TUs was expected because transcription terminates within the ∼3 kb broad promoter-proximal region by a combination of mechanisms (Austenaa et al. 2015, 2021; Estell et al. 2021, 2023; Rouvière et al. 2023). As such, exosome sensitivity here represents post-transcriptional RNA degradation rather than an increase in transcription (Supplemental Fig. S5A). Consistent with this, exosome sensitivity was also observed within TSS-proximal regions of multiexonic TUs; however, there was significantly less exosome-sensitive RNA deriving from the gene bodies of this TU class (Fig. 4B). Finally, exosome-sensitive RNA production was also detectable downstream from some multiexonic TESs. This was somewhat surprising because RNA downstream from the PAS was expected to be largely degraded by XRN2 in a process linked to PAS cleavage (Rodríguez-Molina et al. 2023). We conclude that exosome-sensitive RNAs are abundant within TSS-proximal and, to a lesser extent, TES-proximal regions.

Given our observations at snRNA loci (Fig. 3E), we hypothesized that transcription termination at T-rich elements might also account for at least some exosome-sensitive 3′ ends within mono- and multiexonic loci. Accordingly, we used our RNA 3′-seq data to identify the most common hexameric motifs at exosome-sensitive RNA 3′ ends found within 3 kb upstream antisense and downstream sense of protein-coding gene TSSs. In both cases, T6 was the most common motif in HeLa (Fig. 4C) and HCT116 (Fig. 4D) cells. Again, this did not simply reflect a T bias within the underlying DNA coding strand because the percentage of exosome-sensitive 3′ ends terminating at coding strand Ts exceeded the percentage of Ts found in the genome at these regions (Supplemental Fig. S5B,C). We propose that many promoter-proximal exosome substrates are generated by transcriptional termination at T-tracts/T-rich elements.

Previous data imply that, after transcription initiation, full elongation competence is gradually established as RNAPII accelerates throughout an initial ∼3 kb zone (Jonkers et al. 2014; Fong et al. 2022). Suggestive of this region involving DNA-directed termination, exosome-sensitive T6 termini were most frequent within the first 1 kb proximal to the TSS, after which prevalence decreased with increasing distance to the promoter (Fig. 4E). An example of such promoter-proximal termination over an exosome-sensitive T-tract is provided in Supplemental Figure S5D. Thus, promoter-proximal RNAPII is most sensitive to transcriptional termination over T-rich sequences, and its susceptibility to this mechanism diminishes as it moves into the gene. This implies that mature RNAPII elongation complexes do not terminate at T-tracts. To generally analyze termination at T-tracts across multiexonic TUs, we assayed the exosome or XRN2 sensitivity of 3′ ends deriving from T-tracts found at different regions of the gene body and downstream from the TES. Consistent with the data from Figure 4, C and D, there was substantial exosome sensitivity at T-tracts in the promoter-proximal region (Supplemental Fig. S5E). A more limited level of exosome sensitivity at T-tracts was observed downstream from the TES, and only minor exosome sensitivity was observed at T-tracts within the gene body even though more such motifs were present due to the extended lengths of some genes. XRN2 loss caused no accumulation of RNA terminating over T-tracts over any of these regions. We conclude that termination at T-rich elements is most prevalent promoter-proximally. Plotting previously published RNAPII velocity data (Žumer et al. 2021) revealed that as RNAPII speed increases, the generation of exosome-sensitive 3′ ends declines (Supplemental Fig. S5F). This implies that as elongation complexes mature and accelerate within gene bodies, they become less prone to terminate at T-rich elements.

### DNA-directed and torpedo termination mechanisms are largely independent

**Figure 5.**
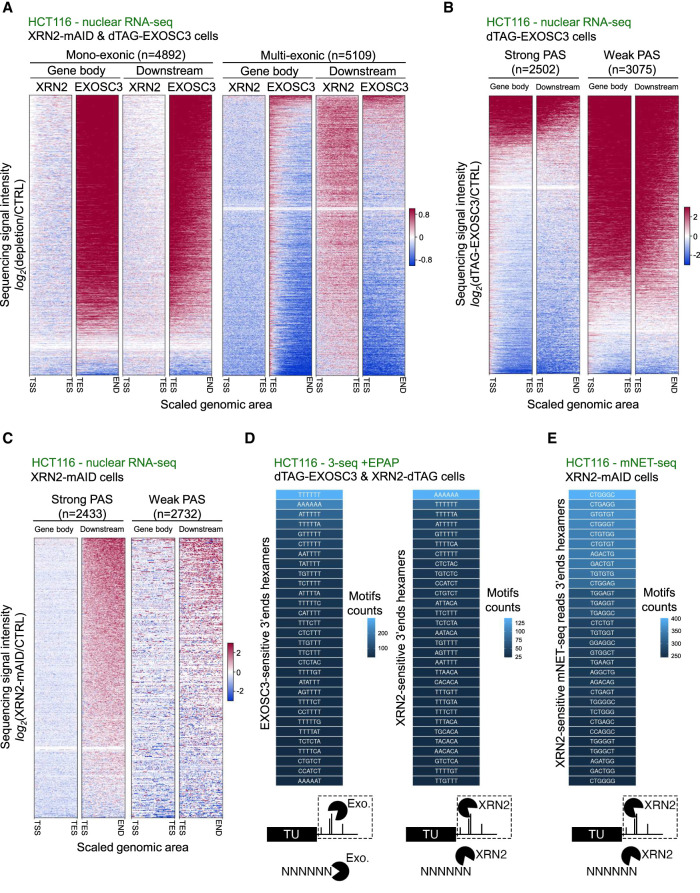
Exosome and XRN2 activities denote separate use of DNA-directed and torpedo termination. (*A*) Heat map analysis as in Figure 4A but showing the log_2_ fold change in nuclear RNA-seq signal intensity between samples obtained after the rapid loss of XRN2 from XRN2-mAID HCT116 cells (XRN2^−^ data from GSE109003) (Eaton et al. 2018) or EXOSC3 from *dTAG-EXOSC3* HCT116 cells (EXOSC3) versus their respective controls. The *left* four heat maps show gene body and downstream regions of monoexonic TUs, and the equivalents at the *right* display the same regions from multiexonic TUs. (*B*) Heat map analysis as in *A* but showing log_2_ change in nuclear RNA-seq signal intensity between −dTAG-EXOSC3 and CTRL samples over the gene body and downstream regions of TUs with predicted strong (*left* two heat maps) or weak (*right* two heat maps) PASs. (*C*) As in *B* but for XRN2-mAID HCT116 cells depleted of XRN2 (Eaton et al. 2018). (*D*) Sequence composition analysis as in Figure 3E but of 3′ ends derived from downstream from multiexonic TU TESs and stabilized by log_2_ change ≥1 following dTAG depletion of EXOSC3 or XRN2 versus their HCT116 cell CTRL. (*E*) Sequence composition analysis as in *D* but of 3′ ends of mNET-seq reads stabilized by log_2_ change ≥1 following XRN2-mAID depletion versus untreated CTRL HCT116 cells. The data are from GSE109003 (Eaton et al. 2018).

XRN2-mediated transcriptional termination is the major mechanism downstream from the PAS. In contrast, DNA-directed termination appears to be most common in promoter-proximal regions. These observations might reflect the two transcriptional termination mechanisms operating in separate contexts. We tested this hypothesis by comparing the exosome and XRN2 sensitivity of transcripts from within and downstream from mono- and multiexonic TUs. To do so, we generated nuclear RNA-seq data from *dTAG-EXOSC3* HCT116 cells and compared them with our previously published nuclear RNA-seq generated after the rapid loss of XRN2-mAID (depleted via the auxin-inducible degron) (Eaton et al. 2018) from HCT116 cells. Consistent with the RNAi-induced depletion of EXOSC3 in HeLa cells (Fig. 4B), rapid EXOSC3 elimination from HCT116 cells revealed that monoexonic RNAs and promoter-proximal transcripts from multiexonic TUs were the most exosome-sensitive, with a milder effect evident downstream from a subset of multiexonic TESs (Fig. 5A). In contrast, monoexonic RNAs and promoter-proximal transcripts from multiexonic TUs were largely unaffected by XRN2 loss. Instead, and as expected due to the widespread torpedo mechanism, RNA deriving from downstream from multiexonic TESs was near-universally stabilized by XRN2 loss. Hence, DNA-directed termination is most prevalent promoter-proximally, whereas torpedo termination is the dominant mechanism downstream from the PAS.

Although RNA cleavage, usually at a PAS, is obligatory for the torpedo termination, it is conceptually unnecessary for a DNA-directed process. We therefore hypothesised that the efficiency of PAS cleavage might dictate the use of either termination pathway downstream from multiexonic gene TESs. To examine this, we used APPARENT2, a machine learning model trained to predict the probability of PAS cleavage for all 23,145 expressed HeLa transcripts (see the Materials and Methods). These were then subdivided into two classes representing those most and least likely to undergo cleavage (assigned as “strong” or “weak” PAS). As expected, protein-coding transcripts were the most enriched biotype with strong PASs, whereas weak signals were primarily assigned to ncRNAs less likely to undergo PAS-dependent processing (Supplemental Fig. S6A; Schlackow et al. 2017; Davidson et al. 2019; Lykke-Andersen et al. 2021). To demonstrate the biological validity of these in silico predictions, we analyzed our published nuclear RNA-seq data obtained after the auxin-induced depletion of CPSF30 (Estell et al. 2021). Because CPSF30 is essential for PAS recognition and consequent transcriptional termination (Clerici et al. 2017; Sun et al. 2018), its depletion was predicted to increase RNA signals beyond all genuinely processed PASs. This was indeed the case for the strong PAS predictions, whereas the predicted weak PASs were mostly unaffected by CPSF30 depletion (Supplemental Fig. S6B).

We then assessed the effect of EXOSC3 or XRN2 loss on nuclear RNA signals upstream of and downstream from weak or strong PASs. Exosome sensitivity was most prevalent upstream of and downstream from the predicted weak PASs, reflecting its known ability to degrade ncRNAs to completion (Fig. 5B). In contrast, XRN2 sensitivity was strongest downstream from efficiently cleaved PASs, and its depletion did not impact RNA levels over gene bodies (Fig. 5C). Although we only had XRN2 depletion data available from HCT116 cells, TT-seq data deriving from siEXOSC3-treated HeLa cells gave a result similar to the dTAG depletion of EXOSC3 from HCT116 cells (Supplemental Fig. S6C). Further leveraging our comprehensive TU annotation in HeLa cells (Lykke-Andersen et al. 2021), we identified T6 as the most common 6 nt motif at 3′ ends stabilized by EXOSC3 loss downstream from weak PASs (Supplemental Fig. S6D). Although T6 was also the most enriched motif downstream from strong cleavage sites, its abundance was ∼10-fold lower than for weak PASs (Supplemental Fig. S6D, note difference in motif count scaling), in line with DNA-directed termination being less frequent here. These analyses reveal that exosome and XRN2 activities are usually used in different locations/circumstances. Specifically, XRN2-dependent termination is associated with PAS cleavage, which is unnecessary for the DNA-directed process.

DNA-encoded information could still contribute to the torpedo mechanism through a scenario in which XRN2 and the exosome cooperatively degrade the fragment downstream from PAS cleavage. If so, XRN2 depletion might also enrich for specific motifs at the terminus of 3′-seq reads downstream from multiexonic TUs, and we therefore compared these over such regions upon EXOSC3 or XRN2 loss in their respective HCT116 degron cells. Consistent with previous analyses, T6 was the top hit after EXOSC3 depletion, with other T-rich regions also enriched (Fig. 5D, left panel). Following XRN2 loss, A6 was the most common 3′ motif, which should be interpreted cautiously because of the used EPAP activity (Fig. 5D, right panel). However, although T6 was ranked second, it was recovered at an approximately fourfold lower frequency than upon EXOSC3 depletion despite the RNA downstream from the PAS being generally more stabilized upon XRN2-depletion. The recovery of XRN2 targeted transcripts with T6 3′ termini might reflect some cooperation between DNA-directed and torpedo termination or the post-transcriptional degradation of RNA already released by DNA-directed termination.

The torpedo mechanism occurs via a “sitting duck” mechanism, whereby RNAPII slows down beyond the PAS and awaits termination by XRN2 (Cortazar et al. 2019). If T-tracts enable XRN2-dependent termination, these “sitting duck” RNAPIIs might be found over such sequences. If so, XRN2 depletion would trap chromatin-associated RNAPII over T-tracts. To test this, we reanalyzed our published mammalian native elongating transcript (mNET)-seq data obtained after the rapid auxin-induced loss of XRN2 in XRN2-mAID cells (Eaton et al. 2018). mNET-seq maps the exact position of RNAPII by sequencing the 3′ end of the RNA residing in its active site (Nojima et al. 2015). We extracted the most frequent XRN2-sensitive 3′ terminal hexamer motifs at the 3′ end of mNET-seq reads downstream from the PAS. Unlike 3′ ends stabilized by EXOSC3 loss and visualized by 3′-seq, these ends were G-rich, and T6 was not among the most abundant motifs (Fig. 5E). This is consistent with previous observations that G-rich elements can act as RNAPII pause sites and facilitate XRN2-dependent termination (Gromak et al. 2006; Sheridan et al. 2019; Fong et al. 2022). Based on this finding, we suggest that RNAPII does not frequently pause/arrest over T-rich elements before XRN2-dependent termination.

### Selected short protein-coding genes use both DNA-directed and torpedo termination

A recent study identified DNA-directed termination downstream from budding yeast protein-coding genes (Han et al. 2023). This was curious, considering our data showing that mammalian DNA-directed termination is most common in promoter proximity. This difference is unlikely to reflect divergent behavior between budding yeast and human RNAPII. Instead, we hypothesized that the distance between the TSSs and DNA-directed termination sites represents a unifying feature. In mammals, this ∼3 kb region corresponds to the promoter-proximal part of the generally long protein-coding genes. However, the same window in budding yeast will include the downstream region of the comparatively much shorter loci. This predicts that short human protein-coding genes might be susceptible to DNA-directed termination.

**Figure 6.**
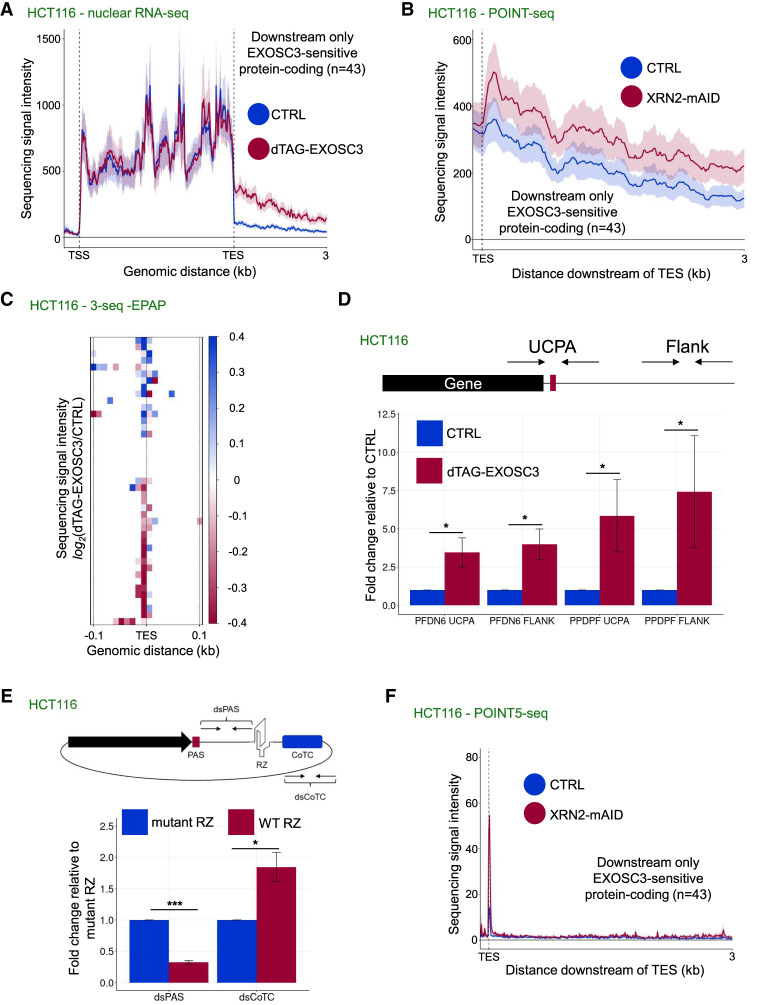
Mutually exclusive use of DNA-directed and torpedo termination downstream from some short protein-coding genes. (*A*) Metaplot of nuclear RNA-seq signal from *dTAG-EXOSC3* HCT116 cells treated or not for 4 h with dTAGv-1. The plot displays signals over 43 protein-coding TUs with a log_2_ change ≥1.5 increase in signal intensity downstream from the TES following EXOSC3 depletion. The *x*-axis shows the gene body region (TSS–TES) and 3 kb of the downstream region. The *y*-axis sequence signal intensity units are RPKM. (*B*) Metaplot as in *A* but for POINT-seq signal derived from *XRN2-mAID* cells treated or not for 4 h with auxin (GSE159326) (Sousa-Luis et al. 2021). The *y*-axis sequence signal intensity units are BPM. (*C*) Heat map showing the log_2_ change in sequencing signal intensity in −EPAP 3′-seq signal samples (dTAG-treated *dTAG-EXOSC3* HCT116 cells vs. CTRL HCT116 cells). The *x*-axis is centered around the TESs of the 43 TUs analyzed in *A* and displays 0.1 kb of respective upstream and downstream regions. (*D*) qRT-PCR analysis of non-PAS cleaved (“UCPA” amplicon) and 3′ flanking (“flank” amplicon) RNAs from the *PFDN6* and *PPDPF* genes. Samples were from dTAG-EXOSC3 cells treated or not with dTAGv-1 for 4 h (dTAG-EXOSC3 and CTRL, respectively). Measured RNA quantities were normalized to GAPDH RNA levels. Mean fold change values were calculated by comparative quantitation and plotted relative to those obtained in CTRL conditions. *n* = 3. Error bars indicate SEM. (*) *P* ≤ 0.05. (*E*) qRT-PCR analysis of RNA derived from human β-globin plasmids containing a hepatitis δ-ribozyme (“WT RZ”) or its inactive mutant (“mutant RZ”) inserted between the PAS and CoTC elements as indicated. Used amplicons were positioned between the PAS and the CoTC (“dsPAS”) and downstream from the CoTC (“dsCoTC”), respectively. Measured RNA quantities were normalized to GAPDH RNA levels. Mean fold change values were calculated by comparative quantitation and plotted relative to those obtained in CTRL conditions. Error bars indicate SEM. *n* = 4. (*) *P* ≤ 0.05, (***) *P* ≤ 0.001. (*F*) Metaplot of published POINT5-seq data from XRN2-AID versus CTRL cells (GSE159326) (Sousa-Luis et al. 2021). The 43 TUs from *A* are shown. The *x*-axis shows the TES and 3 kb of downstream sequence. The *y*-axis sequence signal intensity units are BPM. The only visible XRN2-sensitive 5′ end coincides with the TES (corresponding to PAS cleavage).

To isolate protein-coding genes that undergo DNA-directed transcriptional termination, we used nuclear RNA-seq data to select those whose 3′ flanking RNA was exosome-sensitive. With inclusion criteria of log_2_ fold change ≥1.5 upon dTAG-EXOSC3 loss from HCT116 cells and without any corresponding increase in the gene body, 43 such TUs were selected (Fig. 6A). The latter criterion was important because RNAs from most exosome-sensitive TUs display gene body effects (e.g., Fig. 5A). With only one exception, these 43 TUs were short (∼2.5 kb on average), and most contained a canonical AAUAAA PAS hexamer (Supplemental Fig. S7A). Although their short lengths were more reminiscent of monoexonic TUs, producing primarily exosome-sensitive RNAs, a low level of torpedo termination was evident based on increased POINT-seq signal downstream from their PAS following XRN2 depletion (Fig. 6B; Supplemental Fig. S7B). This finding indicates that the criteria for RNAPII sensitivity to DNA-directed termination in yeast and humans might be similar and depend on RNAPII being relatively close to the TSS.

Our data in Figure 5 indicated that XRN2 does not generally facilitate termination over T-rich elements. However, this might differ at protein-coding genes using the DNA-directed process downstream from their PASs. By seemingly using both interrogated termination mechanisms, these 43 TUs were appropriate to dissect such inter/codependencies. Therefore, we asked whether exosome degradation of 3′ flanking RNA from these TUs was compatible with PAS cleavage, which would be necessary for DNA-directed termination to cooperate with the torpedo mechanism. To do this, we turned to our RNA 3′-seq data obtained without EPAP treatment (to detect naturally cleaved and polyadenylated mRNA). If exosome degradation from a region downstream from the PAS competes with PAS cleavage, we would expect more processed mRNA upon exosome loss. However, EXOSC3 depletion only modestly affected 3′ end processing without a conclusive outcome (Fig. 6C). Furthermore, qRT-PCR analysis of two example transcripts (PFDN6 and PPDPF) with primer pairs spanning their respective PASs (“UCPA”) or 3′ flanks (“flank”) showed significant stabilization of unprocessed RNA upon exosome loss (Fig. 6D). Thus, the exosome activity predominantly targets transcripts that do not undergo PAS cleavage.

The dual EXOSC3 and XRN2 sensitivities of these transcripts and their short TU lengths were reminiscent of our original studies on transcriptional termination using the similarly short (1.8 kb) human β-globin gene as a model (Dye and Proudfoot 2001; West et al. 2004, 2006). β-Globin transcriptional termination requires a PAS as well as a downstream termination sequence, termed the cotranscriptional cleavage (CoTC) element. We originally proposed that RNA transcribed from this terminator was cotranscriptionally cleaved based on its apparent discontinuity and on the dual degradation of the resulting RNA fragment by both the exosome and XRN2 (Dye and Proudfoot 2001; West et al. 2004, 2006). However, the identity of any CoTC endonuclease remained a mystery. Inspired by our findings here, we hypothesised that DNA-directed termination at the CoTC element would generate an exosome substrate. In contrast, the XRN2 entry site would be generated exclusively by PAS cleavage. To test this possibility, we generated a β-globin reporter with a hepatitis δ ribozyme (RZ) or an inactive mutant RZ inserted between the PAS and CoTC elements (Fig. 6E, top panel). RZ cleavage yields an RNAPII-associated cleavage product with an XRN2-resistant 5′OH end, thereby inhibiting the torpedo termination (Stevens and Maupin 1987; Eaton et al. 2020). Hence, if XRN2 degradation initiates solely from the cleaved PAS, RZ cleavage would block its progress and stabilize the downstream RNA. However, if a downstream CoTC event provides an additional XRN2 entry site, the downstream RNA would not accumulate. These constructs were transfected into HCT116 cells, and qRT-PCR was used to assay upstream and downstream RNA levels. The RZ sequence increased readthrough transcription beyond the CoTC element compared with its mutated RZ counterpart (dsCoTC amplicon) (Fig. 6E), suggesting that the CoTC element cannot generate an XRN2 substrate. The upstream RZ cleavage product (dsPAS amplicon) can be degraded (Muniz et al. 2015), explaining the lower level of this species for the WT versus mutant RZ. These data are compatible with CoTC being a DNA-directed terminator. Consistently, CoTC-mediated termination was previously shown to use AT-rich elements, which our results from Supplemental Figure S3E suggest harbor DNA-directed termination capacity.

To test whether PAS cleavages provide the only detectable XRN2 entry sites of the RNAs derived from the 43 TUs displaying strong exosome sensitivity over their 3′ flanks, we analyzed published POINT5-seq data performed after XRN2 depletion (Fig. 6F). Meta-analysis of the 3′ flanking regions showed a single XRN2-sensitive peak, corresponding to the PAS cleavage position. This further suggested that the exosome sensitivity of these RNAs derives directly from termination rather than from the endonucleolytic cleavage of the 3′ flanking RNA. Although our 3′-seq data had low coverage downstream from these genes, some exosome-sensitive T-rich termini were recovered (Supplemental Fig. S7C). Thus, DNA-directed and torpedo termination are mutually exclusive at protein-coding genes that use both mechanisms. Because DNA-directed termination does not require PAS cleavage, it can be used if 3′ end processing fails and when the XRN2 torpedo cannot engage the RNAPII-associated RNA.

Finally, RDH transcripts represent an exceptional class of protein-coding RNAs because their 3′ ends are nonpolyadenylated even though they undergo 3′ end formation using the CPSF73 endonuclease (Marzluff and Koreski 2017). However, like the 43 TUs analyzed here, they are short (<1 kb), and XRN2 has a more limited impact on their transcriptional termination (Fong et al. 2015; Eaton et al. 2018). Thus, DNA-directed termination might play a role here. Consistently, RDH transcripts displayed robust exosome sensitivity downstream from their TESs (Supplemental Fig. S7D). Moreover, T-rich hexamers at EXOSC3 targeted 3′ ends found downstream from RDH TESs were enriched—with more such 3′ ends terminating at Ts compared with the percentage of genomic Ts—in both HCT116 (Supplemental Fig. S7E,F) and HeLa (Supplemental Fig. S7G,H) cells. We conclude that a range of short human protein-coding genes, including RDH TUs, use DNA-directed termination downstream from their TESs.

## Discussion

**Figure 7.**
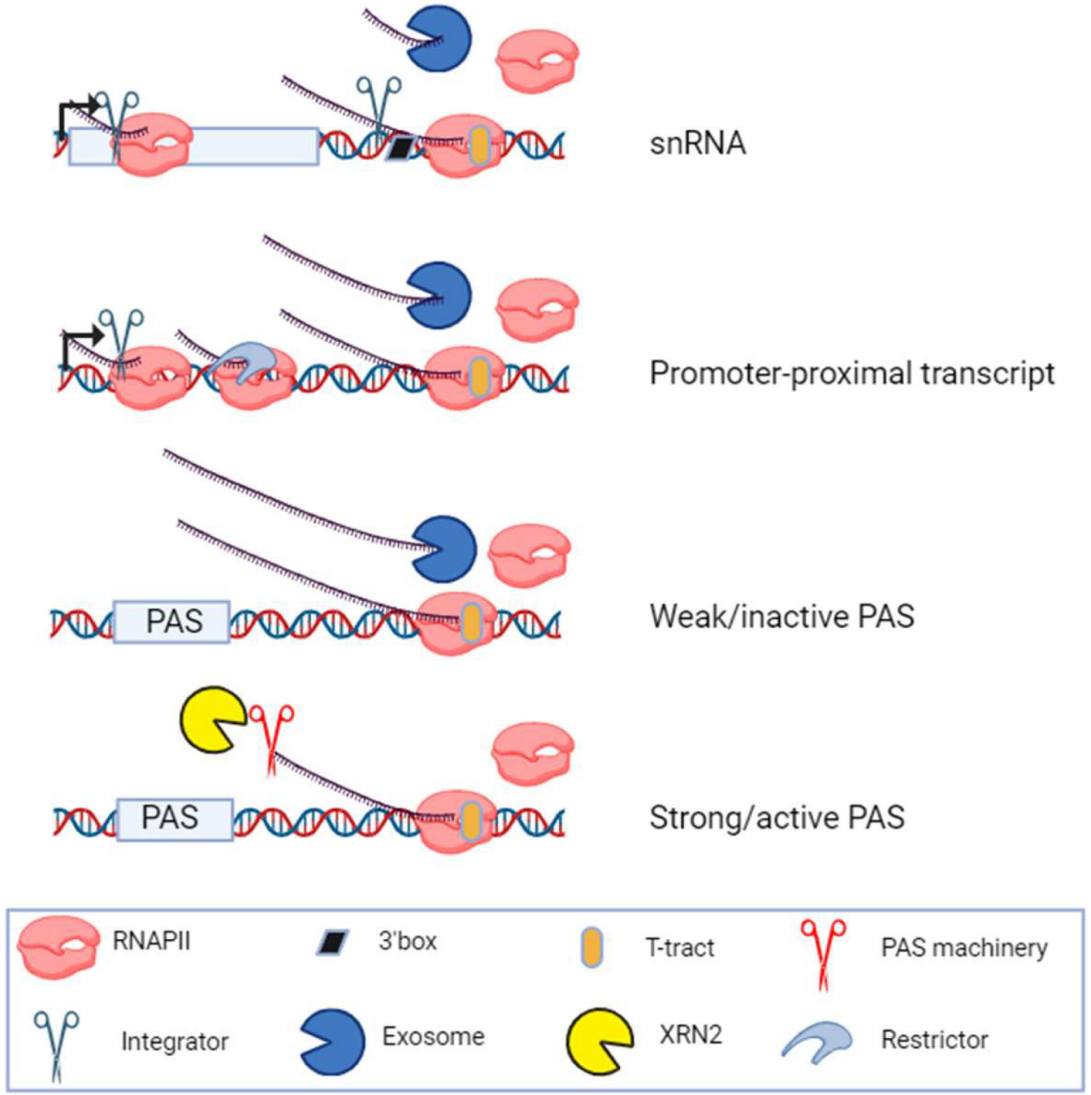
DNA-directed versus torpedo-mediated transcription termination mechanisms. (*Top* two schematics) At snRNA genes, cotranscriptional RNA 3′ box processing by the INT complex precedes but is not required for DNA-directed termination and subsequent exosome degradation. INT also attenuates snRNA transcription, explaining the apparent transcriptional readthrough seen upon its depletion. In promoter-proximal regions, termination can occur via multiple complexes, with INT and Restrictor being prominent examples. However, like for snRNAs, promoter-proximal termination can also occur by a DNA-directed process when RNAPII encounters T-rich elements in the coding strand of DNA. (*Bottom* two schematics) At the ends of multiexonic TUs (like protein-coding genes), efficient PAS cleavage leads to torpedo-directed transcription termination by XRN2 in most cases. Where PAS cleavage is inefficient or fails, DNA-directed termination provides a means of evicting RNAPII from the chromatin template. This might include cases where full elongation competence is not yet established (e.g., on some short protein-coding genes).

The RNA signals required for PAS processing and their connection to transcriptional termination were defined decades ago (Proudfoot 2011). More recently, the ability of the INT complex to elicit RNA cleavage-dependent transcriptional attenuation was also revealed (Elrod et al. 2019; Tatomer et al. 2019; Lykke-Andersen et al. 2021). Instead, prokaryotic RNAP and eukaryotic RNAPIII directly terminate at DNA elements, specifically at T-tracts. Here, we describe the widespread termination of mammalian RNAPII at T-rich elements (Fig. 7). This mechanism is most relevant to RNAPII complexes located promoter-proximally and downstream from a subset of PASs.

Although the importance of sequence in our described DNA-directed mechanism is clear from the enrichment of T-stretches at exosome-sensitive 3′ termini and the termination of reporter plasmid transcription by natural T-rich terminators, we cannot rule out any direct or indirect participation of protein factors. Indeed, although INTS11 is not required for DNA-directed termination, its depletion indirectly induced some readthrough of such elements. Moreover, we cannot exclude the possibility that some snRNA transcription is terminated by the recently described dual endo- and exonucleolytic activities of INTS11 (Fianu et al. 2024) that cannot be separated by the depletion approaches that we used here.

Although we did not analyze the DNA-directed termination of mammalian RNAPII in a purified system, several decades-old studies have addressed this, and the results support our findings. Purified mammalian RNAPII was shown to terminate over endogenous sequences and at bacterial termination elements without an apparent requirement for additional factors (Dedrick et al. 1987; Reines et al. 1987). When mapped, these termination sites indeed coincided with T-tracts or T-rich elements. In these experiments, some T-tracts were ineffective RNAPII terminators, and RNAPII generally terminated less efficiently than prokaryotic RNAP over bacterial termination sequences. This mirrors what happens in vivo because RNAPII does not terminate at every T-tract that it encounters. In contrast, mammalian RNAPIII termination is highly efficient at the first four or more T-tracts in the DNA coding strand. In structures of terminating RNAPIII (Hou et al. 2021), poly-dT in the coding DNA strand orients into the polymerase exit tunnel, where a so-called fork loop stabilizes it. This is proposed to be critical for the DNA-directed termination process. In RNAPII structures (Hou et al. 2021), this exit tunnel is slightly broader, possibly explaining why DNA-directed RNAPII termination is less efficient and can occur at T-rich elements as well as homopolymeric T-tracts. An inefficient response to T-rich elements is presumably important to provide an opportunity to establish full RNAPII elongation competence.

We revealed that INT-dependent 3′ box cleavage serves a processing function at snRNAs that can be uncoupled from transcriptional termination, which instead can occur over T-tracts. This is in contrast to the CPA complex at the PAS, which links mRNA 3′ end processing with transcriptional termination via the activity of XRN2 (Eaton et al. 2018). Initially appearing to contradict multiple observations of snRNA transcriptional readthrough following INT depletion (Baillat et al. 2005; O'Reilly et al. 2014), which we also observed using different experimental approaches, we propose that this increased RNAPII activity is due to INT also functioning to attenuate snRNA transcription. Alleviation of such attenuation following INT depletion increases snRNA transcription over the gene body and downstream region, which can be misinterpreted as a transcriptional termination defect. This explanation predicts INT loss to increase transcription before the 3′ box is encountered, which we observed using TT-seq and POINT-seq approaches and was evident in published TT-seq data (Stein et al. 2022). Although INT has two roles at snRNA loci, elsewhere in the genome, INT presumably only performs its attenuation function because 3′ box consensus elements are inactive. Previous evidence that inactivation of INTS11 recapitulates the effects of its loss on transcription (Wu et al. 2017; Elrod et al. 2019) suggests that these functions require its endonuclease activity.

DNA-directed termination of budding yeast RNAPII was recently described to occur downstream from the PAS (Han et al. 2023). Here, we report its global use by mammalian RNAPII, which is important given the greater complexity of mammalian genes and genomes: They produce thousands of RNAs terminated in this manner, snRNA transcription terminates by this mechanism, and regulated pausing of RNAPII is generally more prevalent in higher eukaryotes (Adelman and Lis 2012). In most cases, and as exemplified at snRNA loci, DNA-directed termination works independently of RNA processing. Consequently, DNA-directed termination and XRN2-dependent termination often work separately downstream from the PAS. When PAS cleavage occurs, transcriptional termination is XRN2-dependent; however, DNA-directed termination is used downstream from weak PASs or when PAS cleavage is absent. At times, XRN2 may terminate RNAPII positioned over T-tracts or could post-transcriptionally degrade RNA after DNA-directed termination. Indeed, the T-tract termination products accumulate following Rat1 depletion from budding yeast, and T-tracts facilitate Rat1-dependent termination in vitro (Han et al. 2023). Supporting our hypothesis that DNA-directed termination downstream from protein-coding genes occurs when PAS cleavage cannot, Han et al. (2023) observed DNA-directed termination after depleting the PAS endonuclease Ysh1.

In contrast to promoter-proximal regions, T-tracts are rarely used for termination within gene bodies. In budding yeast, SPT5 inhibits DNA-directed termination in purified systems and may function similarly in mammals (Han et al. 2023). In mammals, there is a “pausing zone” within 3 kb of the TSS where elongation is slower than in the gene body and premature transcriptional termination is frequent (Supplemental Fig. S5F; Jonkers et al. 2014; Fong et al. 2022). Transcription beyond this region requires SPT5 phosphorylation, and the reversal of this state re-establishes termination competence beyond the PAS (Cortazar et al. 2019). Thus, SPT5 phosphorylation is high where DNA-directed termination is poor and low where it is frequent. We speculate that pausing zones associated with unphosphorylated SPT5 are also efficient termination zones where a DNA-dependent process is common. Other mechanisms that improve elongation, such as telescripting by U1 snRNA (Kaida et al. 2010; Mimoso and Adelman 2023), may also suppress this termination within long introns.

Our early work on transcriptional termination used the human β-globin gene as a model and studied its dedicated CoTC termination element. RNA transcribed from the CoTC sequence was proposed to be cotranscriptionally cleaved and enabled our original discovery of the role of XRN2 in RNAPII termination (West et al. 2004). We hypothesized that cleavage of CoTC RNA provided an entry site for XRN2 to access RNAPII-associated RNA, yet a responsible endonuclease was never discovered. We now suggest that termination occurs directly over the CoTC element, which is compatible with our previous findings that CoTC “activity” generates exosome substrates (Dye and Proudfoot 2001; West et al. 2006). Downstream from protein-coding genes, DNA-directed/CoTC termination may help evict RNAPII that is ill configured for normal 3′ end processing or facilitate termination downstream from weak PASs that are not processed.

We speculate that short human protein-coding genes have more DNA-directed termination downstream from the PAS because some of their RNAPII elongation complexes have insufficient distance to mature and acquire resistance to T-rich elements. In this respect, these genes resemble promoter-proximal transcripts. This fits the idea that elongation competence is established over an initial ∼3 kb window, within which we show that DNA-directed termination is common (Fig. 4E). The much shorter length of budding yeast genes (∼1.5 kb on average) might explain why DNA-directed termination is more general downstream from the PAS compared with humans (Han et al. 2023). Finally, several in vitro studies have claimed that RNAPII transcriptional termination is independent of PAS cleavage and can occur via an allosteric mechanism (Orozco et al. 2002; Zhang et al. 2015). The efficiency of PAS cleavage is typically poor in vitro, and transcription assays are usually performed on short DNA templates. Therefore, the previous observations of PAS-independent termination in such systems may reflect DNA-directed termination of RNAPII that cannot engage the torpedo mechanism due to defective PAS processing.

To conclude, we describe a mode of mammalian RNAPII termination driven by T-rich DNA sequences. Together with the known function of T-tracts in terminating prokaryotic RNAP and eukaryotic RNAPIII, our findings imply a conserved susceptibility to such termination. The maturation of RNAPII elongation complexes provides resistance to this mechanism. Given the need to transcribe for long distances in mammals, this may be why factor-dependent mechanisms have evolved to couple transcriptional termination of RNAPII to RNA processing.

## Materials and methods

### Cell lines and culture conditions

HCT116 and HeLa cells were cultured in Dulbecco's modified Eagle medium (DMEM) supplemented with 10% fetal calf serum and penicillin/streptomycin at 37°C with 5% CO_2_. Transfections were performed using Jetprime (Polyplus). *INTS1-AID* and *dTAG-EXOSC3* cells were generated by CRISPR/Cas9-mediated nonhomologous end joining using 1 µg of an INTS1/EXOSC3-specific guide RNA (gRNA) plasmid and 1 µg of repair PCR product transfected into subconfluent 6 well plates. Media was refreshed 16 h later, and cells were expanded into 100 mm dishes 48 h later. These contained selective media including 10 μg/mL blasticidin (for dTAG-EXOSC3) or 800 μg/mL neomycin/30 μg/mL hygromycin (for INTS1-AID). After 10–14 days, single colonies were selected for screening by PCR for confirmation of genomic insertion. For *INTS1-AID* cells, TIR1 was subsequently integrated into the AAVS1 safe harbor locus (Addgene 72835) using a similar protocol, selecting for puromycin at 1 mg/mL. *XRN2-dTAG* (Estell et al. 2023) and *INTS11-dTAG* (Eaton et al. 2024) cells are described previously. Additional molecules were used at the following concentrations: 500 µM auxin, 1 µM dTAGv-1, and 500 µM 4_S_U.

### Western blotting analyses

One microgram of GFP reporter plasmid was transfected into 6 well plates (∼50% confluent) of unmodified HCT116 parent cells for 16 h. Cells were then lysed in RIPA buffer (150 mM NaCl, 1% NP40, 0.5% sodium deoxycholate, 0.1% SDS, 50 mM Tris-HCl at pH 8, 5 mM EDTA at pH 8) for 20 min on ice. Extracts were clarified by centrifugation at 13,000 rpm for 10 min, retaining the total protein supernatant.

### Antibodies

Antibodies used in this study were anti-GFP (RRID: AB_2749857), anti-Nucleolin (RRID: AB_300442), anti-CPSF73 (RRID: AB_1268249), anti-EXOSC3 (RRID: AB_2278183), anti-XRN2 (RRID: AB_873178), anti-INTS1 (RRID: AB_2127258), anti-HA (RRID: AB_390918), and anti-EXOSC10 (RRID: AB_10990273).

### RNA isolation

Total RNA was extracted using 1 mL of Trizol according to the manufacturer's protocol. RNA was treated with Turbo DNase for 1 h at 37°C. Nuclear RNA was isolated by pelleting cells in PBS, resuspending cells in 5 mL of hypotonic lysis buffer (10 mM Tris at pH 5.5, 10 mM NaCl, 2.5 mM MgCl_2_, 0.5% NP40), and then underlaying with 1 mL of a 10% sucrose cushion. RNA was isolated from this pellet using Trizol.

### qRT-PCR analyses

One microgram of RNA was reverse-transcribed into cDNA using random hexamers and Protoscript II reverse transcriptase (NEB). cDNA was diluted to 50 µL. qRT-PCR was performed using Luna SYBR (NEB) on a Rotorgene (Qiagen). Fold changes were calculated using the ΔCT procedure. Further graphical analysis was achieved in R.

### 4sU labeling and biotinylation

HeLa cell data were published by Lykke-Andersen et al. (2021). For HCT116 cells, two 100 mm culture dishes of cells were labeled with 4_S_U for 5 min. Total RNA was extracted and DNase-treated as described above. For biotin labeling, 5 µg of 4sU-labeled *Drosophila* S2 4_S_U-labeled RNA was spiked into 300 µg of extracted RNA samples and mixed with 10 mM HEPES (pH 7.5), 1 mM EDTA, 10 µg of MTSEA biotin-XX (Biotium), and 1 µL of RNase inhibitor to a final volume of 250 µL. Fifty microliters of dimethylformamide was added, and samples were incubated for 45 min at room temperature in the dark. Samples were made up to 400 µL with water, phenol-chloroform-extracted, and ethanol-precipitated. Pellets were resuspended in 200 µL of wash buffer (10 mM Tris-Cl at pH 7.4, 50 mM NaCl, 1 mM EDTA). One-hundred microliters of µMACS streptavidin microbeads (Miltenyi Biotec) was washed as previously described (Lugowski et al. 2018), once with nucleic acid equilibration buffer (provided) and three times in 100 µL of wash buffer using a µMACS magnetic separator, before the beads were collected in 200 µL of wash buffer off the magnet and mixed with the resuspended RNA and 1 µL of RNase inhibitor. Columns were returned to the µMACS separator and washed three times with 400 µL of wash buffer 1 (10 mM Tris-Cl at pH 7.4, 6 M urea, 10 mM EDTA) prewarmed to 65°C and three times with 400 µL of wash buffer 2 (10 mM Tris-Cl at pH 7.4, 1 M NaCl, 10 mM EDTA). RNA was eluted by four washes with 100 mM dithiothreitol (DTT) dissolved in wash buffer and ethanol-precipitated after incubation for 1 h at −20°C. Trace DTT was removed by precipitating again in 80% ethanol and centrifuging at 13,000 rpm for 10 min.

### 3′-seq

*E. coli* poly(A) polymerase (EPAP) treatment was performed using the poly(A) tailing kit (Thermo) as previously described with some modification (Wu et al. 2020). Three-hundred nanograms of 4_S_U biotinylated RNA was divided into two samples and prepared for EPAP^+^ and EPAP^−^ reactions containing 8 µL of 5× EPAP buffer, 4 µL of 25 mM MnCl_2_, 0.4 µL of EPAP, and 0.4 µL of RNase inhibitor to a final volume of 40 µL with water, replacing EPAP enzyme with water for −EPAP reactions. Samples were incubated for 30 min at 37°C, phenol-chloroform-extracted, and ethanol-precipitated by centrifugation at 13,000 rpm for 10 min. Next, rRNA was depleted using the NEBNext rRNA depletion kit v2 (human/mouse/rat), and libraries were generated using QuantSeq 3′mRNA-seq library preparation kit (Lexogen). Libraries were sequenced on an Illumina NovaSeq6000 (Novogene).

### POINT-seq and POINT5-seq

POINT-seq was performed as previously described (Sousa-Luís et al. 2021). Briefly, confluent 150 mm plates were washed and scraped in PBS and then pelleted before extraction of nuclear RNA as above. Chromatin-associated RNAPII was extracted as follows: Nuclei were resuspended in 100 µL of NUN1 (20 mM Tris-HCl at pH 7.9, 75 mM NaCl, 0.5 mM EDTA, 50% glycerol, 0.85 mM DTT) before being incubated with 1 mL of NUN2 + Empigen (20 mM HEPES at pH 7.6, 1 mM DTT, 7.5 mM MgCl_2_, 0.2 mM EDTA. 0.3 M NaCl, 1 M urea, 1% NP40, 3% Empigen) for 15 min on ice. The subsequent chromatin gel was washed in 10 mL of PBS, resuspended in 250 µL of DNase buffer (10 mM Tris-HCl at pH 7.5, 400 mM NaCl, 100 mM MnCl_2_, 4 mL of RNase inhibitor, 12 µL of Turbo DNase), and incubated for 15 min at 37°C before centrifugation at 13,000 rpm for 10 min. The resulting supernatant was diluted 10-fold in NET2E buffer (50 mM Tris-HCl at pH 7.4, 150 mM NaCl, 0.05% NP-40, 3% Empigen) and precipitated for 1.5 h at 4°C with rotation using 10 µg of anti-RNAPII conjugated to 200 µL of sheep antimouse IgG Dynabeads (Thermo). The beads were washed six times with ice-cold NET2E before RNA was isolated with Trizol. RNA was divided into two samples before processing into POINT and POINT5 libraries as described. POINT-seq libraries were made with the NEBNext Ultra II directional RNA library preparation kit (NEB). POINT5-seq libraries were made with the SMARTer stranded RNA-seq kit (Takara Bio) protocol. Libraries were sequenced on an Illumina NovaSeq6000 (Novogene).

### Nuclear RNA-seq

Nuclear RNA-seq libraries were prepared from DNA-free nuclear RNA using the NEBNext Ultra II directional RNA library preparation kit (NEB) after first depleting rRNA using the NEBNext rRNA depletion kit v2 (human/mouse/rat). Sequencing was performed using an Illumina NovaSeq6000 (Novogene).

### Primer sequences

Primer sequences are listed in Supplemental Table S1.

### Bioinformatics

All bioinformatics methods are detailed in the Supplemental Material.

### GEO accession numbers

New data generated as part of this publication were all HCT116 cell 3′-seq (GSE264499), HCT116 *INTS1-AID* cell POINT5-seq (GSE264716), HCT116 *INTS1-AID* cell POINT-seq (GSE264717), and HCT116 *dTAG-EXOSC3* cell nuclear RNA-seq (GSE264718). Published data were downloaded for *DIS3* PAR-CLIP (GSE64332); *XRN2-AID* POINT-seq and POINT5-seq (GSE159326); INTS11, RRP40, and INTS11xRRP40 siRNA depletion 3′-seq EPAP- and non-EPAP-treated (GSE151919); INTS11 and RRP40 siRNA depletion TT-seq and RNA-seq (GSE151919); XRN2-mAID nuclear RNA-seq and mNET-seq (GSE109003); CPSF30-mAID nuclear RNA-seq (GSE163015); and SPT6-dTAG and RTF1-dTAG DMSO-treated TT-seq and mNET-seq samples (GSE159633).

## Supplemental Material

Supplement 1

Supplement 2

Supplement 3
