# Supplementary material for: DNA-directed termination of mammalian RNA polymerase II
Source: Genes Dev. 2024 Nov-Dec;38(21-24):998–1019. doi: 10.1101/gad.351978.124 (PMC11610936; doi:10.1101/gad.351978.124)
Supplement: Supplement 1 [file SUPPLEMENTAL_MATERIALS_AND_METHODS.pdf]

SUPPLEMENTAL MATERIALS AND METHODS

BIOINFORMATICS

Illumina data pre-processing

Adapters were removed from raw Illumina reads using Trim_galore (https://github.com/FelixKrueger/TrimGalore(Martin 2011)) and aligned to Ensemble GRCh38 using Hisat2 (Kim et al. 2015) (default settings). Aligned reads were next filtered using SAMTools discarding unmapped, poorly mapped (MAPQ < 30), multi-mapped, and if paired-end, discordant reads (Li et al. 2009). Biological replicates were merged using BamTools after PCA plot verification (Barnett et al. 2011). Afterward, merged replicate samples were split by strand using SAMTools, and converted into RPKM normalised single nucleotide resolution bigwigs using DeepTools, scaling the antisense strand coverage by -1 (Ramirez et al. 2014). For +/- EPAP 3'-seq data, the 3' end of the original aligned read was extracted using BedTools (Quinlan and Hall 2010). Bigwigs were additionally scaled using aligned S2 spike-in as a normalisation factor and normalised by RPKM. *INTS1-AID* POINT and POINT5 coverage was normalised to BPM (Bins Per Million). Additionally, for POINT5, the 5' end of the aligned read was extracted, preserving its original directionality before the generation of normalised coverage. $\log_2$ change read coverage files were generated using the bigwigCompare function of DeepTools from single nucleotide strand-specific normalised bigwigs. Bigwigs were visualised using IGV (Robinson et al. 2011).

Metagene analysis

Metagenes were generated using DeepTools computeMatrix from strand-specific normalised read coverage over a curated list of BED intervals with further graphical processing within R. In some cases, line smoothing was applied using the following formula: y ~ s (x, bs = "cs").

snRNA 3' box motif discovery

Starting with a list of HCT116 expressed snRNA genes, derived from untreated nuclear RNA-seq data, we performed MEME motif discovery using the initial 30 nucleotides immediately downstream of annotated snRNA TES. Genome-wide consensus motif scanning was performed using FIMO, extracting BED intervals intersecting core chromosomes only. Further analysis of FIMO box data was performed within R using the ChIPseeker package(Yu et al. 2015).

PolyN termination analysis of +EPAP 3'-seq

PolyT/G/C hexamer sequences were extracted in bed format using the SeqKit locate function and a genomic fasta sequence file. Overlapping polyN hexamer intervals of the same orientation were merged before sub-dividing them into groups based on their overlap to in-house curated snRNAs, multi-exon TU, and mono-exonic PROMPTs interval lists using BedTools intersect. Heatmaps were produced using strand-specific $\log_2$ change (-EXOSC3/+EXOSC3) RPKM normalised 3' end read coverage to each of the interval's list, scaling the interval length to 10 nucleotides and including an upstream and downstream region of identical length.

Identification of genes with exosome hypersensitive 3' flanks

Starting with primary transcript isoforms of protein-coding genes, +/- EXOSC3 nuclear RNA-seq data was used to identify potential CoTC-type terminating transcripts by searching for those that fit the following criteria. The $\log_2$ change of coverage over the gene body remained ≤ 1, and the downstream 3 kb flanking region signal increased by ≥ 1.5 in the -EXOSC3 condition. A final list was then manually curated before further analysis.

Genome Browser views

These were generated using the seqNdisplayR package (Lykke-Andersen et al. 2024).

Definition of TU downstream regions

The downstream area of TUs was defined based on the HeLa annotation (Lykke-Andersen et al. 2021), using custom R scripts. For each strand, the distance between the end of a TU and the gene body of a downstream one was measured. TUs for which this distance was shorter than 100bp or overlapped with the end of chromosomes were filtered out. Over the defined distance, the rtracklayer package was used to measure sequencing coverage for each base of two replicates of RRP40 siRNA depletion TT-seq. A pseudo-count of 1 was then added to this coverage which was then $\log_2$-transformed and averaged between replicates. For each interval previously defined, a mean log coverage over the first 50bp downstream of the TES was measured, and intervals where it was below 2.5 were filtered out. To ensure that the downstream area would not encompass TUs invading downstream ones, an additional filtering was performed requiring coverage to reach 0 for at least 100 consecutive base pairs in the defined intervals. Finally, for each interval, the log coverage was binned by 50bp, and sudden drops of signal between bins were measured. The first position where the signal dropped by more than 2-fold between two consecutive bins was considered the end area. TUs for which such a drop could not be identified were filtered out. Following this procedure, a downstream area was defined for 10569 TUs out of the 23145 originally present in the

annotation. Importantly, this subset of the annotation did not display any bias toward specific biotypes.

Definition of homology between 3'-seq read positions (Figure 1D)

Over the displayed area for each sample, positions with detectable 3'end signal were scored as 1 (independent of signal intensity) and all other positions were scored as 0. This binary distribution was then compared across samples and snRNAs to determine the percentage of homology.

Sequencing signal intensity correlation coefficient computation (Figure 1F)

The deeptools matrix generated by the computeMatrix tool used to generate the associated plot was loaded in R. An average of replicates was then performed for each position and the correlation of sequencing signal intensity (Y-axis) between siCTRL and siINTS11 was measured using the "cor" function with the "pearson" method, both in the "Gene Body" and "Downstream" areas.

Readthrough Index (RI) scoring

To provide an RI, TT-seq coverage was measured either within the whole gene body and 200bp downstream of TESs snRNA loci, or 500bp upstream and downstream of TESs at all other TUs. The ratio of downstream *vs.* upstream signal was then measured at each TU and plotted. Two-sided, paired Mann-Whitney-Wilcoxon tests were performed between samples.

Velocity Measurement

Velocity measurements were performed using published methodology (Zumer et al. 2021). Using control samples, coverage was measured using TTseq and mNET data, and velocity was calculated as the ratio between the intensities of the two.

Definition of exosome/XRN2-sensitive 3' ends

These were defined using a custom R script. Using the previously defined downstream area, sequencing coverage for each base was measured using the rtracklayer package in relevant 3'-seq datasets treated with EPAP. A pseudo-count of 1 was applied at each position and $\log_2$-transformation was performed. For each replicate, we measured the $\log_2$(cov) difference between EXOSC/XRN2 depletion and controls. These $\Delta\log_2$(cov) were then averaged. Stabilised 3'ends were defined as those displaying an average $\Delta\log_2$(cov) above 0.5.

PAS strength prediction

PAS strength prediction was performed using custom R and Python script and the APARENT package (https://github.com/johli/aparent), as in (Wu et al. 2020). For each analyzed TU, the sequence 70bp upstream of the TES was retrieved and searched for the presence of one or more PAS hexamers, according to the ranked list of top 100 hexamers used in humans from (Gruber et al. 2016). When more than one hexamer was identified, the best-ranking one was chosen, and when more than one hexamer with the best rank was identified, the one closest to the position 20bp upstream of the TES was chosen. An area of 205bp placing the beginning of the identified hexamer at position 71 was then defined. For TUs where no hexamer could be identified, the 205bp area was defined placing the TES at position 71. The obtained sequences were then analyzed with the APARENT package, using the 'aparent_all_libs_resnet_no_clinvar_wt_ep_5_var_batch_size_inference_mode_no_drop.h5' model, to predict the cleavage probability by the cleavage and polyadenylation machinery. APARENT provides two scores ranging from 0 to 1, predicting cleavage efficiency based on the PAS hexamer located in position 71 ('narrow score') or anywhere within the 205bp sequence provided ('wide score'). Based on the score's distribution over the HeLa annotation, and the fact that some TUs such as PROMPTs were not expected to get a proper PAS hexamer, TUs with a 'Strong' PAS were defined as those having a 'wide score' above or equal to 0.5 and TUs with a 'Weak'

REFERENCES

Barnett DW, Garrison EK, Quinlan AR, Stromberg MP, Marth GT. 2011. BamTools: a C++ API and toolkit for analyzing and managing BAM files. *Bioinformatics* **27**: 1691-1692.

Gruber AJ, Schmidt R, Gruber AR, Martin G, Ghosh S, Belmadani M, Keller W, Zavolan M. 2016. A comprehensive analysis of 3' end sequencing data sets reveals novel polyadenylation signals and the repressive role of heterogeneous ribonucleoprotein C on cleavage and polyadenylation. *Genome research* **26**: 1145-1159.

Kim D, Langmead B, Salzberg SL. 2015. HISAT: a fast spliced aligner with low memory requirements. *Nat Methods* **12**: 357-360.

Li H, Handsaker B, Wysoker A, Fennell T, Ruan J, Homer N, Marth G, Abecasis G, Durbin R, Genome Project Data Processing S. 2009. The Sequence Alignment/Map format and SAMtools. *Bioinformatics* **25**: 2078-2079.

Lykke-Andersen S, Rouviere JO, Schmid M, Gockert M, Jensen TH. 2024. Protocol for generating customizable and reproducible plots of sequencing coverage data using the seqNdisplayR package. *STAR Protoc* **5**: 102960.

Lykke-Andersen S, Zumer K, Molska ES, Rouviere JO, Wu G, Demel C, Schwalb B, Schmid M, Cramer P, Jensen TH. 2021. Integrator is a genome-wide attenuator of non-productive transcription. *Molecular cell* **81**: 514-529 e516.

Martin M. 2011. Cutadapt Removes Adapter Sequences From High-Throughput Sequencing Reads. *EMBnetjournal* **v17 n1**: 10-12.

Quinlan AR, Hall IM. 2010. BEDTools: a flexible suite of utilities for comparing genomic features. *Bioinformatics* **26**: 841-842.

Ramirez F, Dundar F, Diehl S, Gruning BA, Manke T. 2014. deepTools: a flexible platform for exploring deep-sequencing data. *Nucleic acids research* **42**: W187-191.

Robinson JT, Thorvaldsdottir H, Winckler W, Guttman M, Lander ES, Getz G, Mesirov JP. 2011. Integrative genomics viewer. *Nat Biotechnol* **29**: 24-26.

Wu G, Schmid M, Rib L, Polak P, Meola N, Sandelin A, Jensen TH. 2020. A Two-Layered Targeting Mechanism Underlies Nuclear RNA Sorting by the Human Exosome. *Cell reports* **30**: 2387-2401 e2385.

Yu G, Wang LG, He QY. 2015. ChIPseeker: an R/Bioconductor package for ChIP peak annotation, comparison and visualization. *Bioinformatics* **31**: 2382-2383.

Zumer K, Maier KC, Farnung L, Jaeger MG, Rus P, Winter G, Cramer P. 2021. Two distinct mechanisms of RNA polymerase II elongation stimulation in vivo. *Molecular cell* **81**: 3096-3109 e3098.
