# Supplementary material for: DNA-directed termination of mammalian RNA polymerase II
Source: Genes Dev. 2024 Nov-Dec;38(21-24):998–1019. doi: 10.1101/gad.351978.124 (PMC11610936; doi:10.1101/gad.351978.124)
Supplement: Supplement 3 [file Supplemental_Figures.pdf]

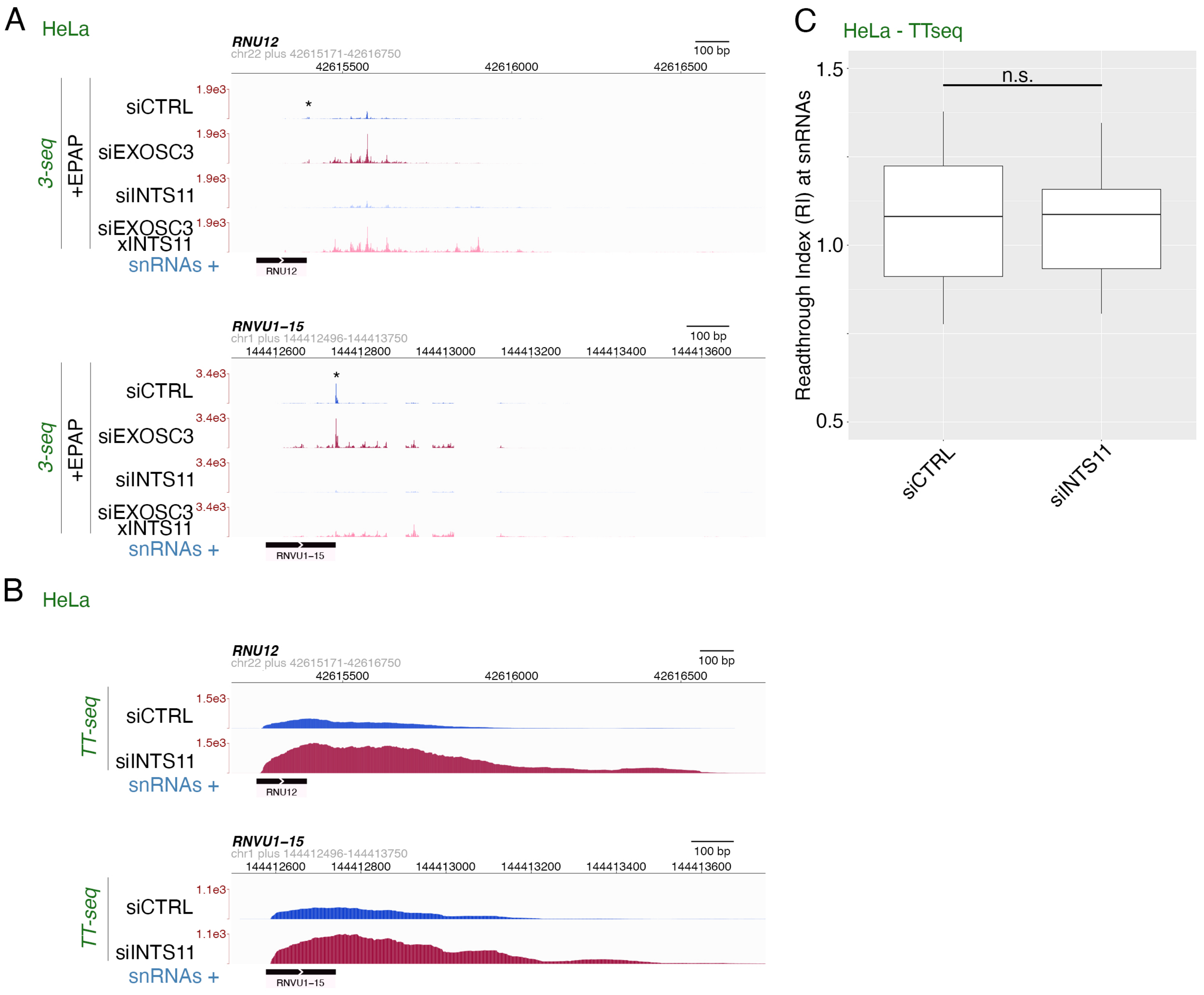
A
HeLa
RNU12
chr22 plus 42615171-42616750
100 bp
42615500
42616000
42616500
3-seq
+EPAP
siCTRL
siEXOSC3
siINTS11
siEXOSC3 xINTS11
1.9e3
*
snRNAs +
RNU12
RNVU1-15
chr1 plus 144412496-144413750
144412600
144412800
144413000
144413200
144413400
144413600
3.4e3
RNVU1-15
B
HeLa
TT-seq
1.5e3
1.1e3
C
HeLa - TTseq
Readthrough Index (RI) at snRNAs
1.5
1.0
0.5
n.s.
siCTRL
siINTS11

Figure S1

SUPPLEMENTAL FIG.S1, related to Figure 1.

A. Genome browser views as in Figure 1B but displaying EPAP data across and downstream of the *RNU12* and *RNVU1-15* TUs. The data are from GSE151919 (Lykke-Andersen et al. 2021)

B. Genome browser view as in (A) but displaying TT-seq data from siCTRL and siINTS11-treated HeLa cells (GSE151919 (Lykke-Andersen et al. 2021)).

C. Box plot comparing the read-through index (RI) at snRNAs in TT-seq samples from siCTRL and siINTS11-treated HeLa cell samples (GSE151919 (Lykke-Andersen et al. 2021)).

A

HCT116 - POINT5-seq

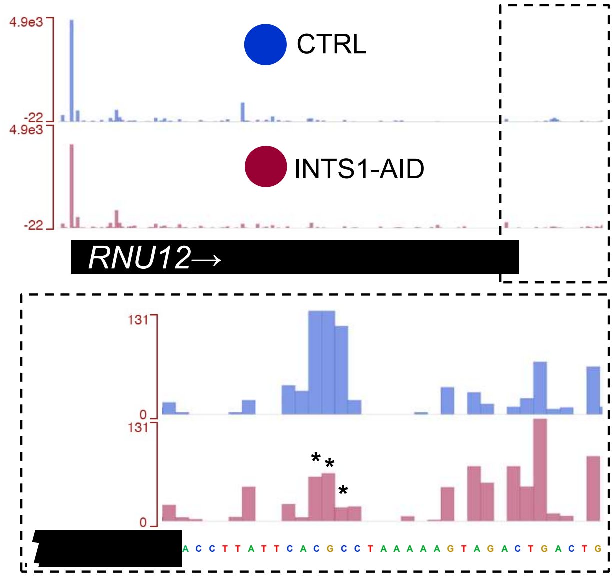

B

HCT116

30 nucleotides

snRNA

Name	p-value	Start		Box motif	
RNVU1-2A	7.65E-08	16	TTTTTTTCTG	GTTTCAAAAATAGAC	
RNU5E-1	7.26E-07	11	TATTTTAAGT	GTTTTAAAAACAGAT	GCGAT
RNU5E-4P	8.63E-07	11	TTATTTGAGC	GTTTTAAAAGTAGAT	GCCCT
RNVU1-6	1.01E-06	11	TATTTTTGT	GTTTTAAAAATAGGT	TTTGT
RNU5D-1	5.04E-06	11	CATCAACGGT	GTTTAAAAATCAGAT	AGAAA
RNU2-63P	5.84E-06	9	AACCATTA	GTGACAAAAACAGGT	TTGTAAT
RNVU1-27	5.84E-06	12	CGCTTTTGTG	GTGCGAATAGTAGAT	GAGC
RNVU1-14	6.56E-06	13	CGCTTTTGTG	GTGCGAATAGTAGGT	GAG
RNVU1-15	6.56E-06	12	TGCTTCTGTG	GTGCGAATAGTAGGT	GAGC
RNVU1-19	6.56E-06	12	TGCTTTTGTG	GTGCGAATAGTAGGT	GAGC
RNVU1-30	7.38E-06	11	GGTTGTTGTC	GTTCTAAAAGTGGAT	TTTGT
RNVU1-29	9.22E-06	10	ATTTTTGTA	GTTTAAAGAACAGTC	TGCACG
RNVU1-3	1.04E-05	12	GATTGCTGTC	GTTTTAAAAGTGGAT	TTTG
RNVU1-28	1.04E-05	11	ATTTTTTGTA	GTTTAAAGAATAGTC	TACAC
RNU12	2.27E-05	12	CCTTATTCAC	GCCTAAAAAGTAGAC	TGAC
RNU5B-1	2.27E-05	11	ATATAAAGCT	GTTAAAAAATCAGA	TGACT
RNVU1-2	4.21E-05	11	ATTTTTTGTA	GTTTCAAGAATACGC	TGTAC
RNVU1-7	8.69E-05	10	AATTTTTGT	AATGAAAAAATAGAC	TCCCCT
RNVU1-31	8.69E-05	10	AATTTTTGT	AATGAAAAAATAGAC	GGCAAG
RNVU1-1	2.84E-04	10	AATTTTGTG	CTGGAAAACGCAGAT	TGTATG
RNU5A-8P	3.25E-04	8	ATCATGT	TTTATAAAAAAAGAC	TTAAAGAG
RNVU1-32	3.49E-04	7	TAAAAA	TTTTTAAAAATAAAT	AAATACATA
RNVU1-22	3.73E-04	11	AGATACTGCA	GTGCAAAACGTGAGC	TCTAC
RNU4-2	4.82E-04	15	ATTTTTAAAA	GTCTAAAGAAAAGGG	T
RNU5A-1	6.57E-04	10	ATATGTGGT	AATCCAACAATAGAA	ATTATT
RNU4-1	7.84E-04	15	ATTTTCTTGC	AGTTGAACAACAGAG	G
RNU2-2P	1.14E-03	8	CTTCGGG	GAGAGAACAACCGTT	GTTTAATG
RNU5E-6P	1.14E-03	12	CATTTTTAAG	TATTAAAAGTTAGGC	AACT

C

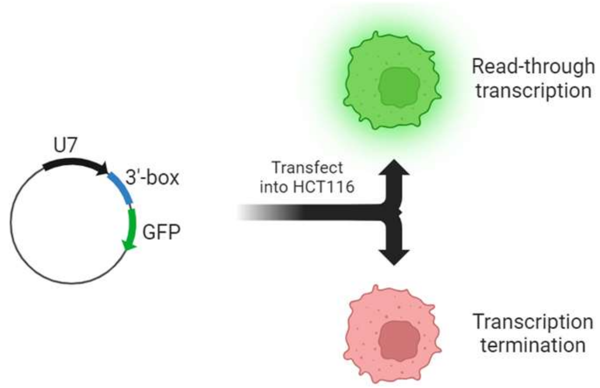

D

HCT116

Name	Box sequence
pWT	GTTGCCAATGATAGAT
pCon	GTTTTAAAAACAGAT
pInv	ATCTGTTTTTAAAAC
pGTT	ACATTAAAAACAGAT
pA5	GTTTTCCCCGCAGAT
pAGA	GTTTTAAAAACCACT

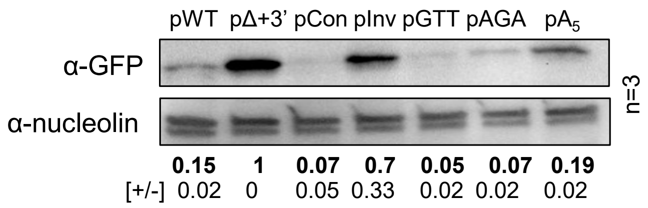

E

HCT116 - POINT5-seq

INTS1-AID cells

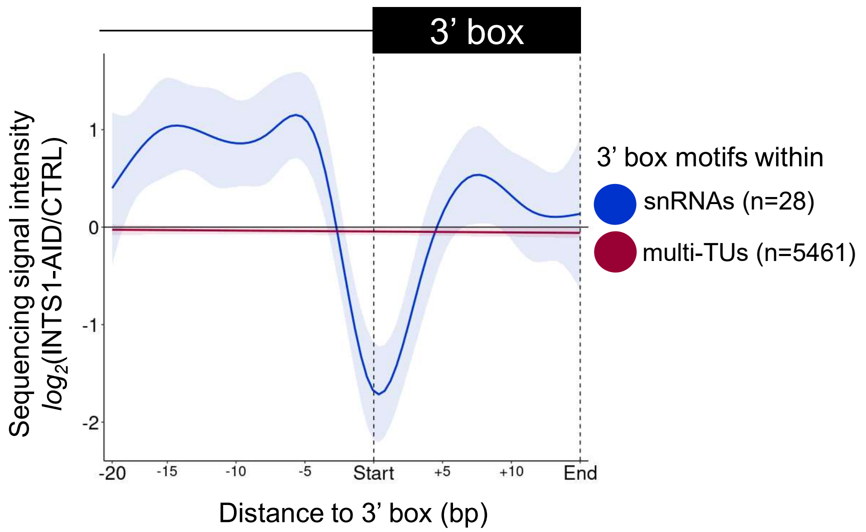

SUPPLEMENTAL FIG. S2, related to Figure 2.

A. Genome browser view as in Figure 2D, but displaying POINT5-seq coverage over the *RNU12* TU.

B. Result of MEME motif consensus over the first 30 nucleotides downstream of the TESs of the indicated Ensembl-annotated expressed snRNAs. The ‘start’ column indicates the number of nucleotides between the TES of each snRNA and the beginning of the 3’ box element (coloured). The first nucleotides downstream of significant POINT5-detectable and INTS1-sensitive cleavage sites are boxed. The p-value represents the confidence for each identified 3’ box motif.

C. Schematic representation of the U7-GFP reporter plasmid (left) and assay (right). If processing occurs at the 3’ box, GFP expression is lost (red cell). Processing failure results in GFP expression (green cell).

D. Western blotting analysis (bottom panel) of HCT116 cells transfected with GFP reporter constructs harbouring the indicated 3’ box motif variants (top panel). The mean relative GFP protein quantities were calculated relative to those obtained in pΔ3’ box samples (reporter variant lacking a 3’ box) after normalising to nucleolin protein levels (n=3, +/- values = SEM).

E. Metaplot of POINT5-seq data from *INTS1-AID* HCT116 cells untreated (CTRL) or treated (INTS1-AID) with auxin. The x-axis covers 20 nucleotides upstream of the start of the 3’ box until the end of the 3’ box element. The y-axis shows the $\log_2$ signal intensity ratio (INTS1-AID/CTRL). The blue line shows data from 3’ box instances at snRNAs and the reduced signal at the start of the 3’ box signifies co-transcriptional processing by INT. The red line shows instances of the consensus 3’ box in the promoter-proximal regions of multi-exonic TUs and is unchanged by INTS1 depletion indicating no detectable INT cleavage activity.

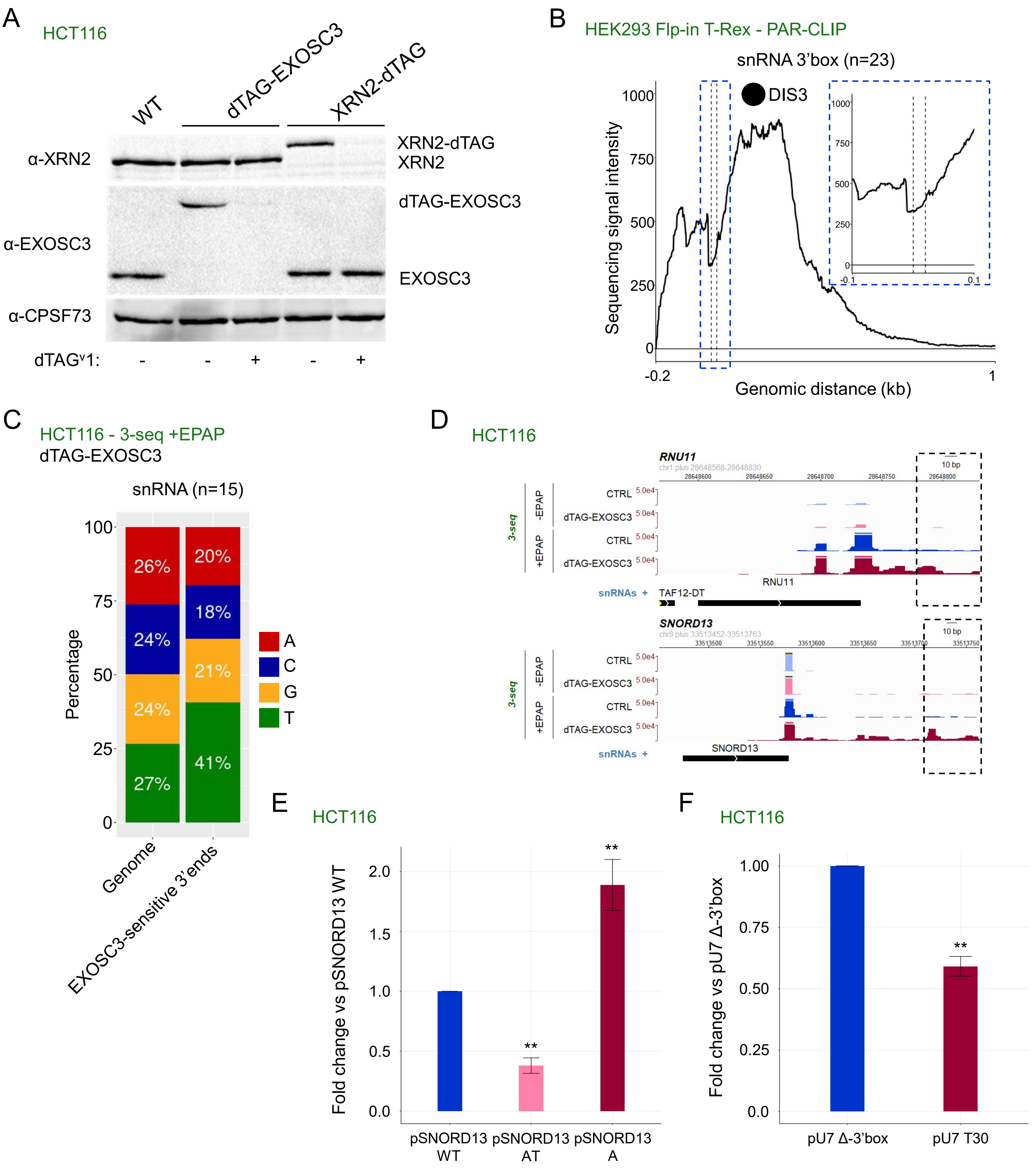
A
HCT116
WT
dTAG-EXOSC3
XRN2-dTAG
α-XRN2
α-EXOSC3
α-CPSF73
XRN2-dTAG
XRN2
dTAG-EXOSC3
EXOSC3
dTAGv1:
- - + - +
B
HEK293 Flp-in T-Rex - PAR-CLIP
snRNA 3'box (n=23)
DIS3
Sequencing signal intensity
Genomic distance (kb)
-0.2
1
0
250
500
750
1000
-0.1
0.1
C
HCT116 - 3-seq +EPAP
dTAG-EXOSC3
snRNA (n=15)
Percentage
0
25
50
75
100
26%
24%
24%
27%
20%
18%
21%
41%
A
C
G
T
Genome
EXOSC3-sensitive 3'ends
D
HCT116
RNU11
chr1 plus 28648568-28648830
10 bp
28648600
28648650
28648700
28648750
28648800
3-seq
-EPAP
+EPAP
CTRL
dTAG-EXOSC3
5.0e4
snRNAs +
TAF12-DT
RNU11
SNORD13
chr8 plus 33513452-33513763
33513500
33513550
33513600
33513650
33513700
33513750
SNORD13
E
HCT116
Fold change vs pSNORD13 WT
0.0
0.5
1.0
1.5
2.0
**
**
pSNORD13 WT
pSNORD13 AT
pSNORD13 A
F
HCT116
Fold change vs pU7 Δ-3'box
0.0
0.25
0.50
0.75
1.00
**
pU7 Δ-3'box
pU7 T30

SUPPLEMENTAL FIG. S3, related to Figure 3.

A. Western blotting analysis of HCT116, *dTAG-EXOSC3,* and *XRN2-dTAG*, cells treated, or not, for 4hr with dTAG-v1 as indicated beneath the blot. CPSF73 was probed as a loading control. Homozygous tagging of EXOSC3 and XRN2 was evident from the slower migration of the dTAG-EXOSC3 and XRN2-dTAG species, which were depleted following dTAGv-1 treatment.

B. Metaplot of DIS3 PAR-CLIP data (GSE64332 (Szczepinska et al. 2015)) across the 3' box region of 23 snRNAs. The x-axis covers the 3' box (black dashed lines) and sequences encompassing 0.2kb upstream and 1kb downstream. The y-axis sequence signal intensity units are RPKM. The zoomed area (blue dashed box) highlights the 3' box and the immediate increase in the downstream PAR-CLIP signal.

C. Histogram showing the % of each nucleotide in the genomic sequence downstream of snRNA TESs ("Genome") compared to the % of each nucleotide at the 3' end of EPAP-treated 3'-seq reads over the same region that were stabilised by $\log_2$ change ≥1 in dTAGv-1 treated *dTAG-EXOSC3* HCT116 cells *vs.* CTRL HCT116 cells ("EXOSC3-sensitive 3' ends").

D. Genome browser views of 3'-seq data obtained from -/+EPAP-treated RNA samples isolated from dTAG-treated dTAG-EXOSC3 cells or HCT116 CTRL cells. The *RNU11* and *SNORD13* TUs and 200 nucleotides of the downstream region are shown. The dashed boxes highlight clusters of 3'-seq reads that are EPAP-dependent and stabilised by dTAG-EXOSC3 depletion.

E. qRT-PCR analysis of GFP mRNA from cells transfected with WT pSNORD13 or its derivatives with T-tracts substituted by AT- or A-tracts. Measured RNA quantities were normalised to RNA levels from a co-transfected β-globin plasmid. Mean fold-change values were calculated by comparative quantitation and plotted relative to those obtained in WT pSNORD13 samples. n=5. Error bars = SEM. ** denotes p≤0.01.

F. qRT-PCR analysis of GFP mRNA as in E but from cells transfected with pΔ3' box or an equivalent plasmid (pT30) containing a homopolymeric T-tract. n=6. Error bars = SEM. ** denotes p≤0.01.

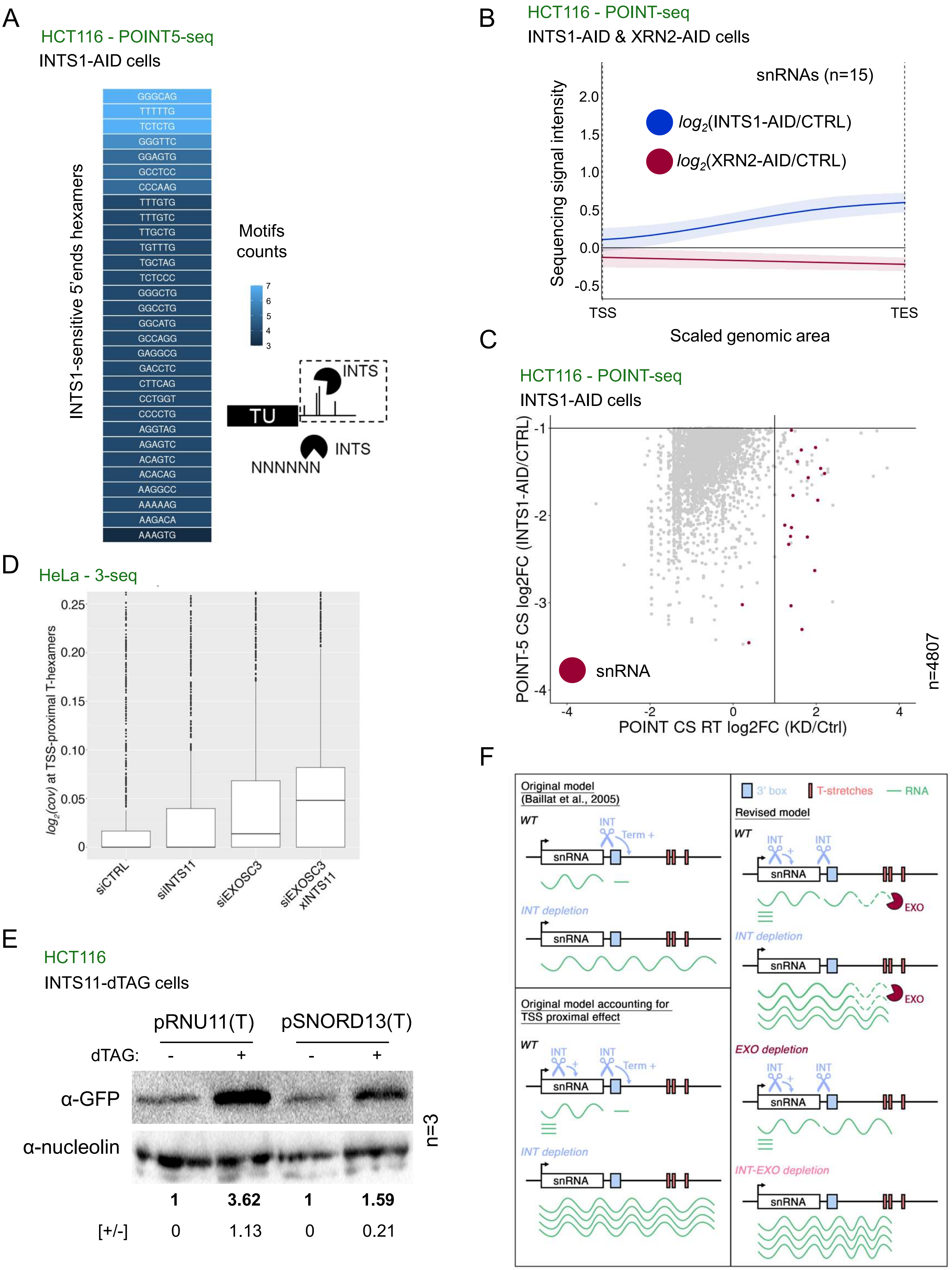
A
HCT116 - POINT5-seq
INTS1-AID cells
INTS1-sensitive 5'ends hexamers
GGGCAG
TTTTTG
TCTCTG
GGGTTC
GGAGTG
GCCTCC
CCCAAG
TTTGTG
TTTGTC
TTGCTG
TGTTTG
TGCTAG
TCTCCC
GGGCTG
GGCCTG
GGCATG
GCCAGG
GAGGCG
GACCTC
CTTCAG
CCTGGT
CCCCTG
AGGTAG
AGAGTC
ACAGTC
ACACAG
AAGGCC
AAAAAG
AAGACA
AAAGTG
Motifs counts
7
6
5
4
3
TU
INTS
INTS
NNNNNN
B
HCT116 - POINT-seq
INTS1-AID & XRN2-AID cells
snRNAs (n=15)
log2(INTS1-AID/CTRL)
log2(XRN2-AID/CTRL)
Sequencing signal intensity
2.0
1.5
1.0
0.5
0.0
-0.5
TSS
TES
Scaled genomic area
C
HCT116 - POINT-seq
INTS1-AID cells
POINT-5 CS log2FC (INTS1-AID/CTRL)
-1
-2
-3
-4
snRNA
n=4807
-4
-2
0
2
4
POINT CS RT log2FC (KD/Ctrl)
D
HeLa - 3-seq
log2(cov) at TSS-proximal T-hexamers
0.25
0.20
0.15
0.10
0.05
0
siCTRL
siINTS11
siEXOSC3
siEXOSC3 xINTS11
E
HCT116
INTS11-dTAG cells
pRNU11(T)
pSNORD13(T)
dTAG:
-
+
-
+
α-GFP
α-nucleolin
n=3
1
3.62
1
1.59
[+/-]
0
1.13
0
0.21
F
Original model (Baillat et al., 2005)
WT
INT
Term +
snRNA
INT depletion
snRNA
Original model accounting for TSS proximal effect
WT
INT
INT
Term +
snRNA
INT depletion
snRNA
3' box
T-stretches
RNA
Revised model
WT
INT
INT
snRNA
EXO
INT depletion
snRNA
EXO
EXO depletion
INT
INT
snRNA
INT-EXO depletion
snRNA

SUPPLEMENTAL FIG. S4, related to Figure 3

A. Sequence composition of the most common hexameric motifs found immediately upstream of POINT5-seq mapped 5' ends located in the region ≤4kb downstream of snRNA TESs and showing sensitivity (log_2 change ≥1) to INTS1-AID depletion from HCT116 cells (see schematics). The heat scale shows the motif count. Low count numbers indicate background signal. Importantly, T6 motifs were not identified suggesting that they are not sites of Integrator cleavage.

B. Metaplot of POINT5-seq data of RNA from *INTS1-AID* HCT116 cells untreated (CTRL) or treated (INTS-AID) with auxin. The plot represents 15 snRNAs separated from their neighbouring TUs by at least 5kb. The x-axis shows gene body regions (excluding the TSS). The y-axis represents log_2 signal intensity ratio (INTS1-AID/CTRL and XRN2-AID/CTRL). XRN2-AID POINT5-seq data are from (GSE159326 (Sousa-Luis et al. 2021)).

C. Scatter plot showing the TSS-proximal log_2 POINT5-seq signal ratio (INTS1-AID/CTRL) (y-axis) as a function of promoter-proximal (within 1kb of the TSS) transcription levels expressed as log_2 POINT-seq signal ratio (INTS1-AID/CTRL) (x-axis). snRNAs are highlighted in red. There were few INTS1-sensitive signals elsewhere.

D. Box plot quantifying read-through index (RI) at snRNAs in 3'-seq experiments from siCTRL-, siINTS11-, siEXOSC3- or siINTS11xEXOSC3-treated HeLa cells (GSE151919 (Lykke-Andersen et al. 2021)).

E. Western blotting analysis of GFP expression following transfection of *INTS11-dTAG* HCT116 cells with p^{term}RNU11, or p^{term}SNORD13 containing reporter constructs. Quantifications below the western membrane show relative GFP expression levels relative to those of the pΔ3' box reporter. n=3 (+/- values = SEM) following normalisation to nucleolin protein levels. INTS11 was depleted for 14hr.

F. Revised model for INT functions in snRNA maturation and transcription. The original model posited essential coupling of INT-mediated 3' box cleavage and snRNA transcriptional termination. We now show that INT affects snRNA transcription through both transcriptional attenuation and cleavage at the 3' box. The latter is unnecessary for transcriptional termination, which can occur in a DNA-directed manner. The RNA products of this mechanism are degraded by the nuclear exosome. The INT attenuation function indirectly affects DNA-directed termination by controlling the flux of RNAPII through snRNA. This model explains the exosome-sensitivity of snRNA precursors, the previously observed effects of INT on snRNA transcription, and the additive effects of co-depleting INT and EXOSC3 on snRNA precursor accumulation. Some snRNA transcriptional termination may employ sequential endo- and exonuclease activity of INTS11, which cannot be assayed in our experiments.

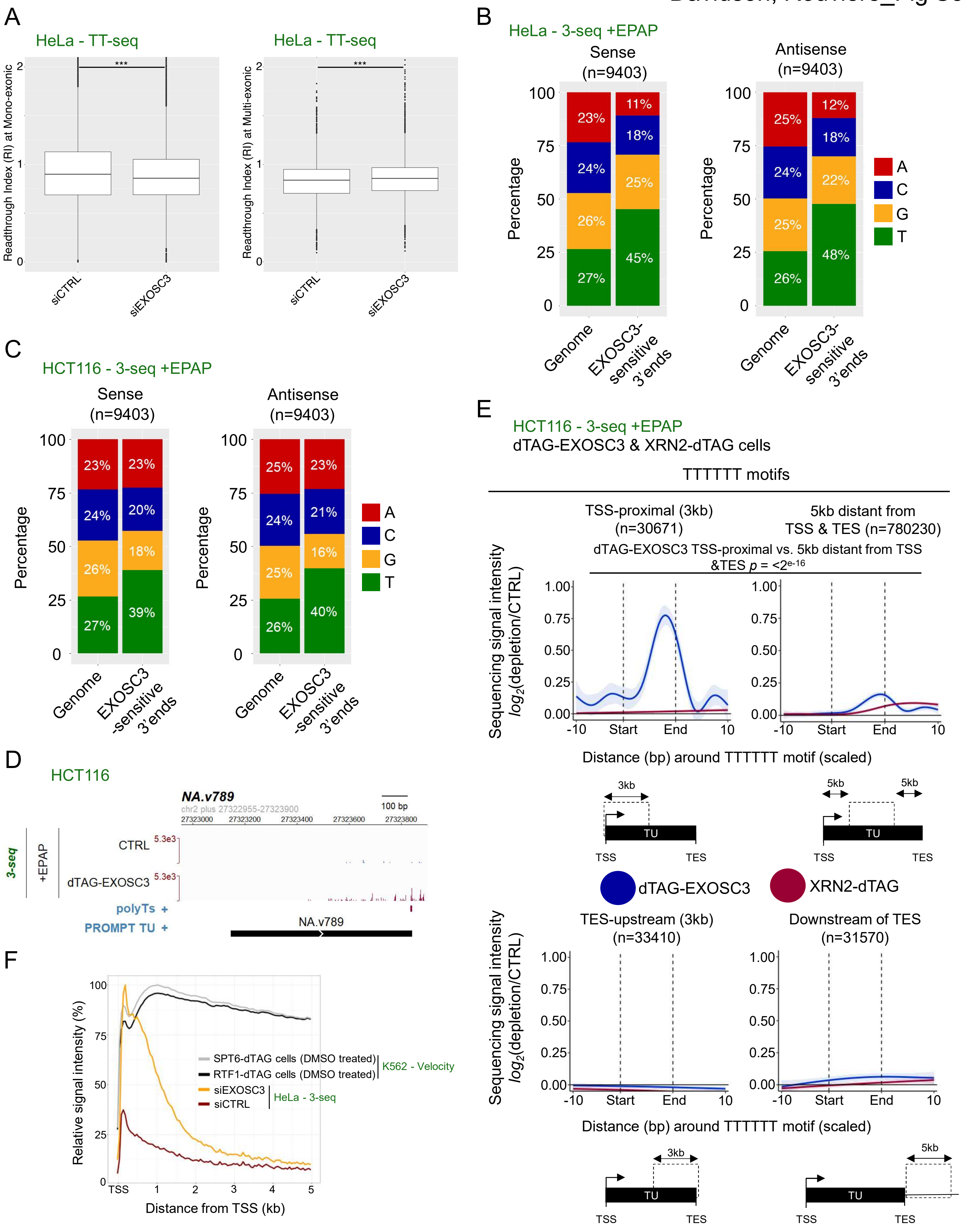
A
HeLa - TT-seq
Readthrough Index (RI) at Mono-exonic
Readthrough Index (RI) at Multi-exonic
***
siCTRL
siEXOSC3
B
HeLa - 3-seq +EPAP
Sense (n=9403)
Antisense (n=9403)
Percentage
Genome
EXOSC3-sensitive 3'ends
A
C
G
T
C
HCT116 - 3-seq +EPAP
D
HCT116
NA.v789
chr2 plus 27322955-27323900
100 bp
3-seq
+EPAP
CTRL
dTAG-EXOSC3
polyTs +
PROMPT TU +
E
HCT116 - 3-seq +EPAP
dTAG-EXOSC3 & XRN2-dTAG cells
TTTTTT motifs
TSS-proximal (3kb) (n=30671)
5kb distant from TSS & TES (n=780230)
dTAG-EXOSC3 TSS-proximal vs. 5kb distant from TSS &TES p = <2e-16
Sequencing signal intensity log2(depletion/CTRL)
Distance (bp) around TTTTTT motif (scaled)
TES-upstream (3kb) (n=33410)
Downstream of TES (n=31570)
dTAG-EXOSC3
XRN2-dTAG
TSS
TES
TU
F
Relative signal intensity (%)
Distance from TSS (kb)
SPT6-dTAG cells (DMSO treated)
RTF1-dTAG cells (DMSO treated)
K562 - Velocity
siEXOSC3
siCTRL
HeLa - 3-seq

SUPPLEMENTAL FIG. S5, related to Figure 4.

A. Box plot of data from Figure 4A, but displaying the read-through index at mono- and multi-exon TUs in TT-seq performed in siCTRL- and siEXOSC3-treated HeLa cells (GSE151919 (Lykke-Andersen et al. 2021)).

B. Histogram as in Figure S3C but showing the genomic sequence within 3kb sense (left) and antisense (right) of TSSs compared to the % of each nucleotide at the 3' end of EPAP-treated 3'-seq reads over the same region that were stabilised by $\log_2$ change ≥1 in siEXOSC3- vs siCTRL treated HeLa cells ("EXOSC3-sensitive 3' ends"). The data are from GSE151919 (Lykke-Andersen et al. 2021)

C. Histogram as in (A) but for data obtained in dTAGv-1 treated *dTAG-EXOSC3* HCT116 cells *vs.* CTRL HCT116 cells.

D. Genome browser view of a PROMPT TU showing +EPAP 3'-seq coverage in CTRL HCT116 cells and dTAG-EXOSC3 HCT116 cells treated with dTAGv-1 (4hr). The location of the T-tract is indicated by the red bar under the lower track.

E. Metaplots showing the $\log_2$ sequencing intensity change in +EPAP 3' end signals, comparing dTAG-treated *dTAG-EXOSC3* or *XRN2-dTAG* HCT116 cell samples with CTRL HCT116 cell samples. The plot displays multi-exonic TUs divided into four regions as indicated below: 3kb TSS-proximal, gene body (omitting the 5kb TSS- and TES-proximal regions, 3kb upstream of the TES, and 5kb downstream of the TES. Each plot displays signal changes across T≥6 sequences and includes 10 nucleotides upstream and downstream.

F. Graph plotting RNAPII velocity data (Zumer et al. 2021) and the production of exosome-sensitive 3' ends (via 3'-seq from GSE151919 (Lykke-Andersen et al. 2021)) over the first 5kb of multi-exonic TUs. The y-axis shows relative signal intensities expressed as % of the maximum.

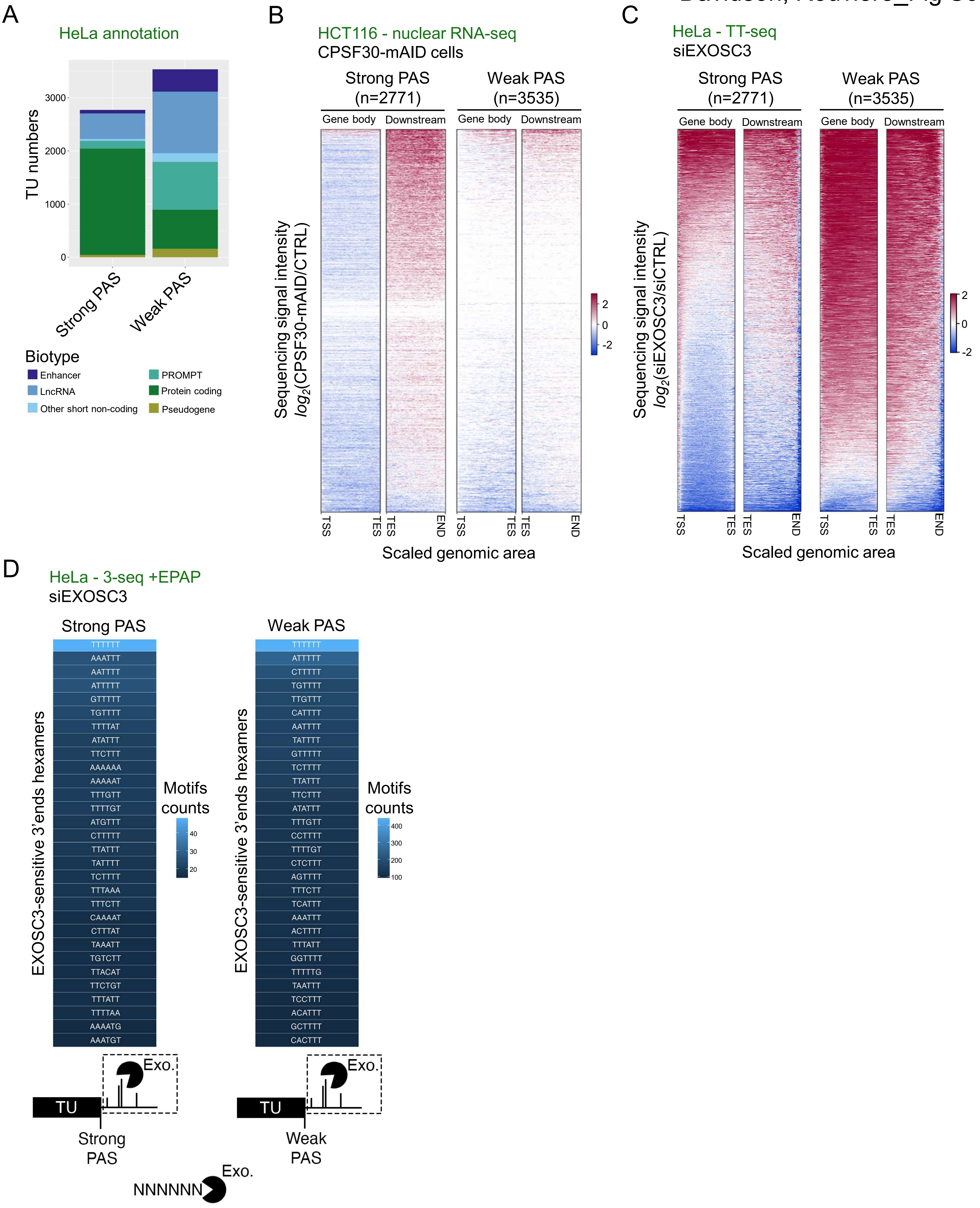
A
HeLa annotation
TU numbers
3000
2000
1000
0
Strong PAS
Weak PAS
Biotype
Enhancer
LncRNA
Other short non-coding
PROMPT
Protein coding
Pseudogene
B
HCT116 - nuclear RNA-seq
CPSF30-mAID cells
Strong PAS (n=2771)
Weak PAS (n=3535)
Gene body
Downstream
Sequencing signal intensity
log2(CPSF30-mAID/CTRL)
2
0
-2
TSS
TES
END
Scaled genomic area
C
HeLa - TT-seq
siEXOSC3
Strong PAS (n=2771)
Weak PAS (n=3535)
Gene body
Downstream
Sequencing signal intensity
log2(siEXOSC3/siCTRL)
2
0
-2
TSS
TES
END
Scaled genomic area
D
HeLa - 3-seq +EPAP
siEXOSC3
Strong PAS
TTTTTT
AAATTT
AATTTT
ATTTTT
GTTTTT
TGTTTT
TTTTAT
ATATTT
TTCTTT
AAAAAA
AAAAAT
TTTGTT
TTTTGT
ATGTTT
CTTTTT
TTATTT
TATTTT
TCTTTT
TTTAAA
TTTCTT
CAAAAT
CTTTAT
TAAATT
TGTCTT
TTACAT
TTCTGT
TTTATT
TTTTAA
AAAATG
AAATGT
EXOSC3-sensitive 3'ends hexamers
Motifs counts
40
30
20
Weak PAS
TTTTTT
ATTTTT
CTTTTT
TGTTTT
TTGTTT
CATTTT
AATTTT
TATTTT
GTTTTT
TCTTTT
TTATTT
TTCTTT
ATATTT
TTTGTT
CCTTTT
TTTTGT
CTCTTT
AGTTTT
TTTCTT
TCATTT
AAATTT
ACTTTT
TTTATT
GGTTTT
TTTTTG
TAATTT
TCCTTT
ACATTT
GCTTTT
CACTTT
Motifs counts
400
300
200
100
Exo.
TU
Strong PAS
Weak PAS
NNNNNN
Exo.

SUPPLEMENTAL FIG. S6, related to Figure 5.

A. Histogram showing the proportion of the indicated RNA biotypes classified by APPARENT2 as having a strong or a weak PAS. All TUs with a 3' end defined by TT-seq data after EXOSC3 depletion from HeLa cells were included (see Materials and Methods).

B. Heatmap analysis as in Figure 5B but showing the $\log_2$ change in nuclear RNA-seq signal intensity following CPSF30-mAID depletion from HCT116 cells (CPSF30-mAID *vs.* CTRL from GSE163015 (Estell et al. 2021)). Note that CPSF30 loss increases signal downstream of strong but not weak PASs. CPSF30 loss also reduces the gene body signal upstream of strong PASs - a well-described consequence of rapid CPA factor loss (Eaton et al. 2020; Cugusi et al. 2022).

C. Heatmap analysis as in (B) but for TT-seq data derived from siEXOSC3- and siCTRL-treated HeLa cells (GSE151919 (Lykke-Andersen et al. 2021)).

D. Sequence composition analysis as in Figure 5D, but of 3' ends beyond strong or weak PASs and stabilised by $\log_2$ change ≥1 following siEXOSC3 *vs*. siCTRL treatment of HeLa cells (GSE151919 (Lykke-Andersen et al. 2021)).

Davidson, Rouviere_Fig S7

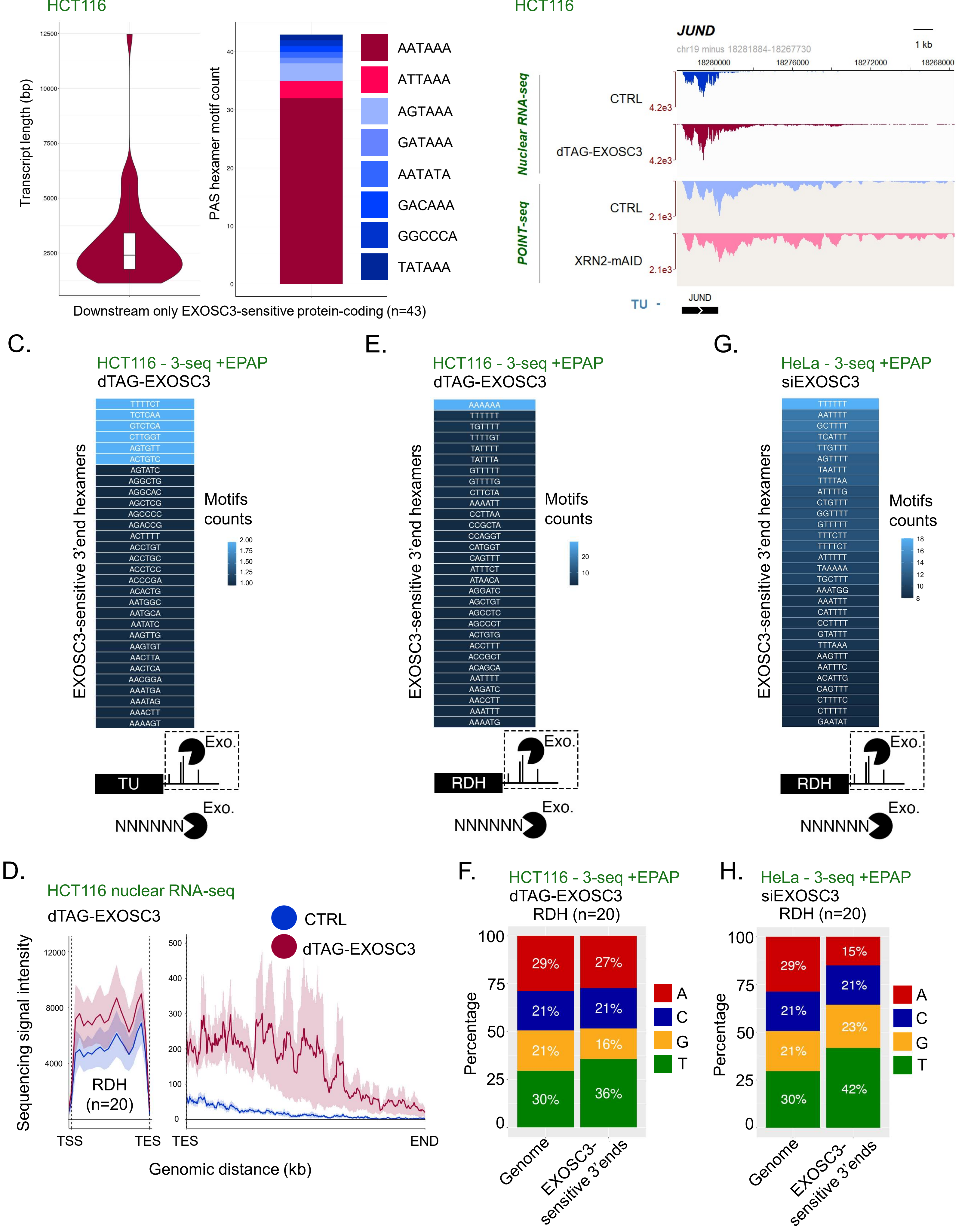

SUPPLEMENTAL FIG. S7, related to figure 6.

A. Left: Violin plot showing TU length distributions for the 43 examples studied. The y-axis shows transcript length in nucleotides. Right: PAS hexamer motif counts for the 43 TUs.

B. Genome browser view of *JUND* (one of the 43 TUs, harboring EXOSC3-sensitive 3' flanking RNA). Nuclear RNA-seq- (top two tracks) or POINTseq- (bottom two tracks) signals from CTRL HCT116 cells or degron derivatives depleted of dTAG-EXOSC3 (nuclear RNA-seq) or XRN2-mAID (POINT-seq - GSE159326 (Sousa-Luis et al. 2021)) are shown. The y-axis scales are RPKM.

C. Sequence composition analysis as in Figure 5D, but of 3' ends deriving from downstream the TESs of the 43 studied TUs.

D. Metaplots of nuclear RNA-seq signal from *dTAG-EXOSC3* HCT116 cells treated, or not, for 4hr with dTAGv-1. The left and right plots display signals over RDH gene bodies and downstream of RDH TESs, respectively. The y-axis sequence signal intensity units are RPKM.

E. Sequence composition analysis as in (C), but of 3' ends deriving from downstream RDH TESs. Note that although A6 is the top motif, this should be interpreted cautiously due to the employed EPAP activity. Other highly enriched motifs are T-rich.

F. Histogram as in Figure S3C but showing the % of each nucleotide in the genomic sequence downstream of RDH TESs ("Genome") compared to the % of each nucleotide at the 3' end of EPAP-treated 3'-seq reads over the same region stabilised by $\log_2$ change ≥1 in dTAGv-1 treated *dTAG-EXOSC3* HCT116 cells *vs.* CTRL HCT116 cells ("EXOSC3-sensitive 3' ends").

G. Sequence composition analysis as in (E), but displaying signals derived from siEXOSC3 *vs.* siCTRL-treated HeLa cells (GSE151919 (Lykke-Andersen et al. 2021)).

H. Histogram as in (F) but displaying samples derived from siEXOSC3 *vs.* siCTRL-treated HeLa cells.
